# Effects of a depression-focused internet intervention in slot machine gamblers: A randomized controlled trial

**DOI:** 10.1371/journal.pone.0198859

**Published:** 2018-06-08

**Authors:** Lara Bücker, Julia Bierbrodt, Iver Hand, Charlotte Wittekind, Steffen Moritz

**Affiliations:** 1 Department of Psychiatry and Psychotherapy, University Medical Center Hamburg-Eppendorf, Hamburg, Germany; 2 Ambulatory Healthcare Center Falkenried, Hamburg, Germany; TNO, NETHERLANDS

## Abstract

**Background:**

Problematic and pathological gambling have been linked to depression. Despite a high demand for treatment and negative financial consequences, only a small fraction of problematic and pathological gamblers seek professional help. The existing treatment gap could be narrowed by providing low-threshold, anonymous internet-based interventions. The aim of the present study was to examine the acceptance and efficacy of an online-intervention for depression (“Deprexis”) in a sample of problematic and pathological slot-machine gamblers. We hypothesized that the intervention group would show a greater reduction in both depressive and gambling-related symptoms compared to a wait-list control group.

**Method:**

A total of 140 individuals with self-reported gambling and mood problems were randomly allocated either to the intervention group or to a wait-list control group. After 8 weeks, all participants were invited for re-assessment. The Patient Health Questionnaire - 9 (PHQ-9) served as the primary outcome assessment. Problematic gambling was measured with the Pathological Gambling Adaptation of Yale-Brown Obsessive Compulsive Scale (PG-YBOCS) and the South Oaks Gambling Screen (SOGS). The trial is registered with the German Registry for Clinical Studies (DRKS00013888).

**Results:**

ITT analyses showed that the intervention led to a significant reduction in depressive symptoms as well as gambling-related symptoms compared to the control group, with moderate to strong effect sizes. PP analyses failed to yield significant results due to high rates of non-completion and limited statistical power. Moderator analyses indicated that Deprexis was particularly beneficial in reducing problematic gambling for those scoring high on baseline gambling-related symptoms and for those who gamble due to loneliness.

**Discussion:**

Results of the present study suggest that Deprexis might be a useful adjunct to traditional interventions for the treatment of problematic gambling. The potential of internet-based interventions that are more targeted at issues specific to gambling should be evaluated in future studies.

**Trial registration:**

German Registry for Clinical Studies DRKS00013888.

## Introduction

For many people worldwide, gambling at slot machines represents an innocuous leisure activity. At the same time, a considerable proportion of gamblers loses control over gambling behavior and enters into a downwards spiral of psychological, financial and social problems [1–3]. In the 5^th^ Edition of the Diagnostic and Statistical Manual of Mental Disorders [4], gambling problems have been moved from the Impulse-Control Disorders to the Substance Use section, which has been refuted by some experts [5,6]. “Gambling disorder” according to DSM-5 has replaced the term “pathological gambling” and is defined as enduring and bothersome gambling behavior that leads to clinically significant impairment or distress. There is disagreement on the definition of “problem gambling”; several models each define the term differently [7]. While one definition claims that problem gambling denotes a subclinical form of gambling disorder that includes every form of gambling that leads to harmful consequences or difficulty in any area of functioning [7,8], others categorize gamblers in one or the other of the two categories based on the number of DSM-criteria endorsed and thereby draw a more clear distinction between problematic and pathological gambling [9]. In the present article, the terms “problem gambling” and “problematic gambling” are used as general expressions to describe any condition in which gambling leads to various forms of harm and dysfunction. The average prevalence rate for gambling disorder has been estimated at 2.3%, ranging internationally from 0.5% to 7.6% [10]. Although there is a large variety of games (e.g. roulette, blackjack, poker, bingo, sports betting etc.), gambling at slot machines is regarded as the most addictive form of gambling, underlined by the fact that up to 80% of those seeking treatment report this form of gambling [1,11]. Despite the negative consequences and the existence of effective treatments [for a meta-analysis see [12]] about 90% of problem gamblers do not seek help or discontinue treatment [13]. The small number of problem gamblers who seek help are mainly driven by major life crises; that is, they wait until the problems are too severe and burdensome to deny or suppress [14]. Barriers to seek treatment include the urge to solve the problems by oneself, lack of knowledge about (affordable) treatment possibilities, problem denial, and shame [15,16].

### Comorbid depression in gambling disorder

93.6% of pathological gamblers have at least one lifetime Axis I comorbid disorder [9], which complicates treatment. Major depression is one of the most prevalent comorbid disorders (29.9%) among individuals with gambling disorder [17]. Yet, the exact relationship between gambling and depressive symptoms remains unclear. Some of those affected appear to engage in gambling activities to reduce negative emotions and distract themselves from problems in their daily lives. For others, depressive symptoms might develop as a consequence of gambling-induced financial and social crises [7]. Because depressive symptoms worsen the outcome of gambling treatment and vice versa, and comorbid depression significantly raises the risk of suicide [18,19], depressive symptoms should represent a core target in the treatment of gambling.

### Types of problematic gambling

There have been multiple suggestions for categorizing gamblers into specific subtypes depending on gambling motivations and indicated treatments [for an overview see [20]]. The multifactorial pathways model by Blaszczynski and Nower (2002) proposes three different subtypes of problem gamblers. The first one is defined as “behavioral conditioned” problem gamblers who show no signs of premorbid pathology or emotional problems before developing the problematic gambling behavior. The subjective excitement and arousal become associated with playing slot machines, and with repetition, they become classically conditioned to the gambling environment. The second subtype is labeled “emotionally vulnerable” problem gamblers, who have experienced mood problems, problematic coping skills as well as unfavorable social backgrounds and family situations prior to developing the problematic gambling behavior. This type of problem gambler seeks escape in gambling activities mainly to reduce negative emotions. The third type is described as the “antisocial-impulsive” problem gambler who shares all characteristics of the second type but also shows high levels of antisocial and impulsive behaviors or personality traits. A psychopathological-behavioral model of “social vs. escape/avoidance vs. presuicidal gambling” and its consequences for cognitive behavioral therapy (CBT) has been proposed by Hand (1998).

The present study is concerned with the second type, escape seekers, characterized by premorbid depressive symptoms and poor coping and problem solving skills. Depressive symptoms seem to precede gambling problems in more than 70% of the cases [21]. Gamblers with depressive symptoms typically prefer slot machines over other types of gambling [22]; slot machine gambling may reduce negative emotions by inducing dissociation through the repetitive character of this type of gambling. During this trance-like state, called “slot machine zone”, the gambler completely immerses into the game and neglects outside events [23].

### Online interventions for depressive symptoms

Online interventions, mostly based on CBT, have proven to be an anonymous, feasible, cost-efficient and effective addition to existing treatment options for various mental disorders [24,25] and could therefore be provided to narrow the existing treatment gap. Over the last decade, increasingly sophisticated programs have evolved yielding effect sizes comparable to face-to-face interventions [26]. Especially for depressive symptoms, a large body of evidence substantiates the efficacy of online interventions [27–29]. In meta-analyses, unguided online interventions for depression show small-to-medium effect sizes compared to controls, stable over follow-up periods of 4–12 months [30,31]. The program investigated in this study, called “Deprexis”, has proven to be effective in the treatment of primary [32–39] and secondary depression [40,41] in various randomized controlled trials [for a meta-analysis see [42]]. Against an emerging bulk of evidence for the efficacy of online interventions in the treatment of depressive symptoms and first evidence on feasibility and efficacy of online intervention in problem gamblers (see next subsection), we assume that online-interventions could be a meaningful addition to existing treatment options and a foot-in-the-door for those who do not want to seek formal treatment.

### Online interventions for addiction problems

For substance related addictions, there is also a growing body of evidence for the efficacy of online interventions [43], particularly for alcohol use disorders [44] and cigarette smoking [45,46]. CBT appears to be the most effective psychotherapeutic treatment for gambling related disorders [47] and a number of studies have begun to examine the feasibility and efficacy on *guided* CBT-based online interventions in problem gamblers. While these yield promising results [48,49] there is a lack of empirical research on unguided interventions. Whereas guided interventions generally outperform unguided interventions regarding efficacy and treatment adherence (see above), a study on the online treatment of alcohol problems found that therapeutic guidance did not improve intervention outcomes, and notably, most participants denied contacts [50]. The first study comparing unguided to guided interventions found similar effects in problem gamblers; the group with guidance showed a significantly higher dropout rate, while there was no significant difference in symptom improvement between groups. The authors inferred that guidance could have negative effects on gamblers who had not sought help before [51]. Unguided interventions based on automated personal feedback on gambling behavior and psychoeducation about cognitive distortions have proven feasible, perceived as helpful by problem gamblers, and significantly reducing gambling behavior and problems [52–55]. One study found that a brief psychoeducational cognitive intervention targeting gambling-related cognitions was able to reduce the erroneous beliefs, but not the intention to gamble per se [56]. Two RCTs on online delivered CBT have been published to this date. One trial could not detect significant effects of either unguided or guided CBT-based online treatments compared to a control condition among problem online poker gamblers [51]. Another RCT found significant gains on gambling severity, gambling urge, depression, anxiety and stress as well as quality of life [57]. These findings and the growing research efforts reflected by two recently published study protocols [58,59] showed that online interventions for problem gamblers are increasingly considered a feasible complement to conventional treatment or an alternative when face-to-face treatment is either not available or rejected.

### Aim of the present study

The present study is part of a larger project investigating the effectiveness of two different computer based interventions/trainings in problematic gambling. Data and results of the other sub-study are presented elsewhere (Wittekind et al., submitted). The primary aim of this study was to examine the efficacy of the internet intervention Deprexis in treating comorbid depressive symptoms in problematic and pathological slot-machine gamblers. Furthermore, based on the well-established strong ties between depression and gambling problems, we explored whether a reduction of depressive symptoms would be accompanied by a decline in pathological gambling. We expected a larger reduction of both, depressive and gambling related symptoms, in the Deprexis group compared to a wait-list control group.

## Methods

### Recruitment

Participants were recruited between May 2014 and June 2016. Invitations to the trial, including a short study description and a web-link to the baseline survey, were posted in numerous gambling- and addiction-related internet forums, on problem gambling information websites, and social networks (e.g., Facebook groups and information pages). We also ran an online recruitment campaign via Google AdWords, leading persons looking for keywords like “gambling-addicted”, “gambling-addiction help” or “slot-machine addicted” to a sub-webpage of our working group where information as well as the study invitation were presented. Moreover, study flyers were sent to self-help groups, regional counseling centers and psychiatric clinics, and were provided in gambling halls. Additionally, we advertised in several German newspapers. No financial reimbursement was offered for study participation. Participants received free access to the online program either directly after randomization or after finishing the reassessment after eight weeks. In addition, all completers received further self-help material at no cost upon completion of the training (e.g., manual about progressive muscle relaxation and mindfulness).

### Baseline assessment

The trial was set up as an online study using questback^®^ (www.unipark.com/de). The rationale and the procedures of the study were explained on the introductory pages of the survey. We obtained an online informed consent for each participant in accordance with regulations by the ethics committee of the German Psychological Society (DGPs). The survey proceeded with the following sections: demographic and psychopathological information, clinical history (e.g., current treatments, use of medication and self-help, psychiatric diagnoses). Subsequently, different questionnaires were applied (see section “Questionnaires”). Finally, participants were asked to provide an e-mail address and a personal code which was generated according to the guidelines recommended by the ethics committee. The average time for completing the baseline survey was 31 minutes.

The ethics committee of the German Society for Psychology (*Deutsche Gesellschaft für Psychologie*, DGPs, ID: SM 012014_2) has reviewed and approved of this study on March 3^rd^2014. The trial is registered at the German Registry for Clinical Studies DRKS (registration number: DRKS00013888). Before the start of recruitment, organizational changes delayed the registration process. In order to avoid further delays, the study was registered retrospectively. As the study involved human subjects, it was conducted in accordance with the Declaration of Helsinki (1964).

### Inclusion and exclusion criteria

Inclusion criteria were self-reported problem or pathological gambling with slot machines, along with subjective feelings of sadness and desperation, as well as age between 18 and 65 years. Acute suicidality (assessed by one specific item of the PHQ-9) and a lifetime diagnosis of a bipolar or psychotic disorder led to immediate study exclusion. In-/exclusion was carried out automatically based on self-report in the baseline online survey. Furthermore, participants who stated not having answered the survey honestly at the end of the study were excluded. Participants who fulfilled any exclusion criterion were immediately led to a finish page containing an explanation for study exclusion and emergency phone numbers for people in need of immediate psychological support. To prevent excluded persons from repeated enrolment in the survey, participants were blocked from re-access by means of “cookies”.

### Treatment allocation

**Fig 1:**
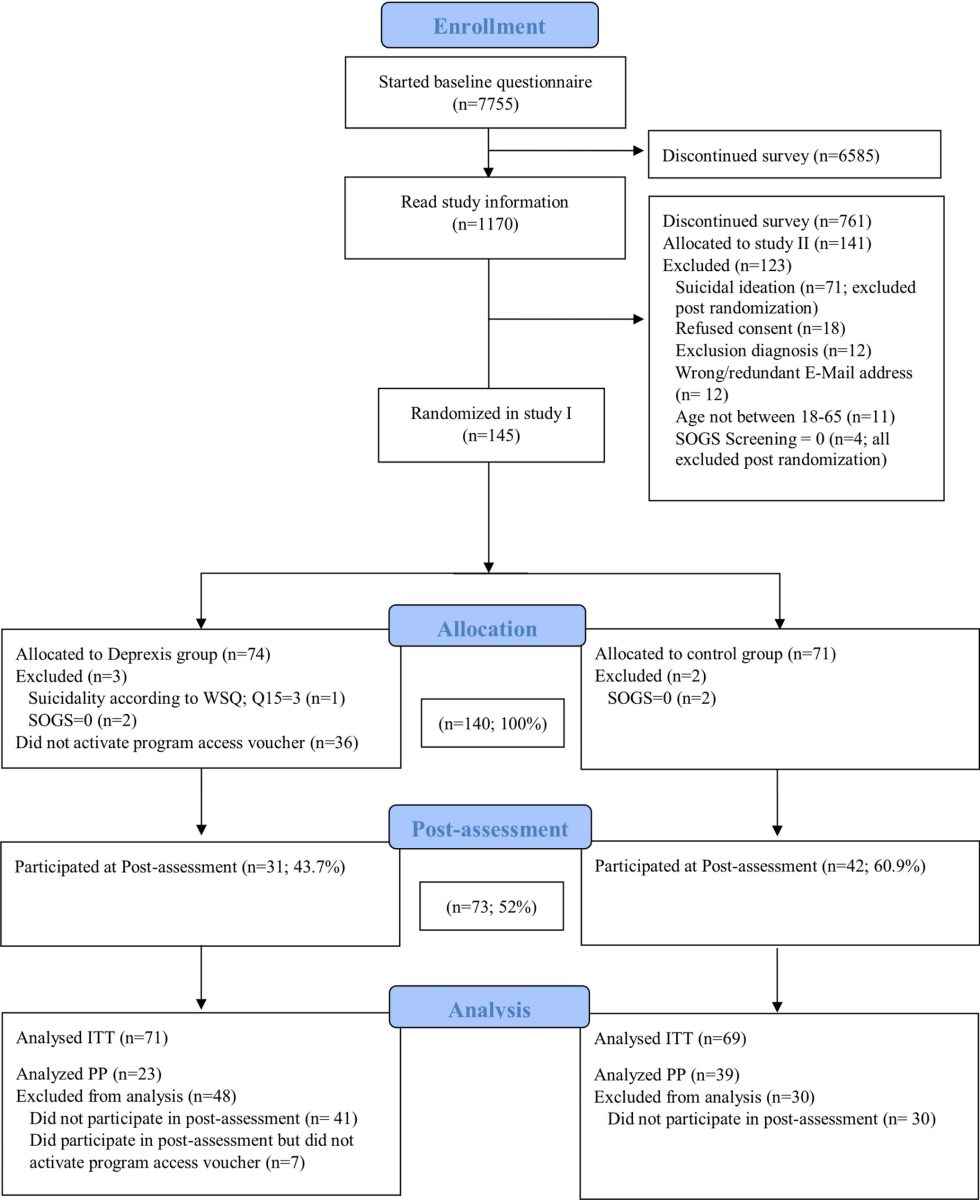
Flowchart of study selection.

This study was part of a larger project investigating the effectiveness of an internet intervention (Deprexis) and an online training program (retraining) in problem gamblers. Initially, a four-arm study was planned. Before the start of the recruitment, we decided to split the four-arm study into two separate studies, each with one intervention and one control group. This was done because the two interventions pursue different goals and therefore necessitate different primary and secondary outcomes. Data on the other study, which evaluated a training program (retraining) by means of the Approach-Avoidance Task [60] will be presented in a seperate paper (Wittekind et al., submitted). Participants who completed the baseline survey were randomly allocated to either Deprexis or the no intervention control group in a pseudo-random order (based on the date and time of the baseline assessment). The randomization sequence was generated with the computer software Research Randomizer [61]. The random allocation rule was 1:1; participants were evenly randomized across conditions. Allocation was concealed for the person (second author) enrolling participants to the study. After finishing the baseline assessment, the experimental group received an e-mail with a registration code for the Deprexis program, whereas the control group received the information that they will get access to the program upon completion of the post-assessment after eight weeks. All participants were informed that they could contact the study team for questions at any time and were allowed to use any form of treatment during the study period, including medication and psychotherapy. The first participant was enrolled in the study on April 25^th^2014, the last participant enrolled completed the post survey on August 23^rd^2016. Fig 1 shows a flowchart of the study selection.

### Intervention

The intervention was the internet intervention Deprexis [for a detailed description see [32,42]]. The program targets depressive symptoms and consists of ten modules with therapeutic content plus one summary module, addressing essential components of evidence-based depression treatment, broadly based on cognitive-behavioral therapy and its third wave. Main topics covered are behavioral activation, cognitive modification, interpersonal and problem solving skills, and relaxation methods, complemented by acceptance and mindfulness techniques as well as positive psychology. The topics are presented in an interactive format, in which the content and order of presentation are dynamically tailored to user requirements using simulated dialogues. The user is presented some information and can then select one of several response options indicating, for instance, approval, the need for further information, or skepticism. Subsequent content is then tailored to the user’s responses and preferences. Each module can be completed in 10–60 minutes, depending on the user’s speed and need for further information. Deprexis can be used on mobile devices and desktop computers.

### Post-assessment

Eight weeks after the baseline assessment, participants were sent an e-mail that included an invitation for the post-assessment. All participants in the treatment condition were invited to participate in the post-assessment regardless of how frequently they had used Deprexis. Initially, participants were asked to enter the same e-mail address and personal code they had used in the baseline survey in order to match pre- and post-data. The post-assessment contained the same psychopathology questionnaires as the baseline assessment (see section “Questionnaires”). Additionally, participants in the intervention group were asked for their subjective appraisal of the intervention. After confirmation that all questions had been answered honestly, participants were thanked for their participation and links were provided to download manuals teaching progressive muscle relaxation and mindfulness. Average time for completing the post-assessment was 25 minutes. Five reminder e-mails were dispatched if participants did not respond to the first invitation. The control group received the access code to Deprexis following the post-assessment.

### Questionnaires

Participants were asked to complete the following questionnaires at pre- and post-assessment.

#### Web Screening Questionnaire (WSQ)

The WSQ [62] is a brief online self-report screening instrument to capture common mental disorders. The questionnaire screens for the following mental disorders: depressive disorders, alcohol abuse/dependence, general anxiety disorder, posttraumatic stress disorder, social phobia, panic disorder, agoraphobia, specific phobia, obsessive compulsive disorder, and suicide risk. The questionnaire has been evaluated as a valid screening instrument with values for sensitivity ranging between .72–1.00 and for specificity between .44–.77 [62]. The WSQ was assessed only at baseline.

#### Patient Health Questionnaire - 9 items depression module (PHQ-9)

The primary outcome measure was the PHQ-9 [63,64], a self-rating measure of depressive symptom severity. The psychometric properties of the PHQ-9 are very good, with high internal consistency [Cronbach's α = .86–.89; (64)]. Its sum score ranges from 0 to 27, with scores from 0–4 indicating none or minimal depression, 5–9 mild depression, 10–14 moderate depression, and 15–27 severe depression. Clinically significant improvement on the PHQ-9 can be defined as a decline of five points [65].

#### South Oaks Gambling Screen (SOGS)

We used the SOGS [66] as a 20-items self-report measure to screen for engagement in gambling activities and gambling related problems. It is the most used measure of gambling difficulties internationally [67]. In the present study the questionnaire was used as screening instrument for pathological gambling. The internal consistency is acceptable (Cronbach's α = .69); its convergent validity is higher showing strong correlations with DSM-IV criteria for pathological gambling (*r* = .72, .57, *P* < .001) [68]. The SOGS was initially developed based on DSM-III criteria, but continues to correlate with recent versions of the DSM [69].

#### Pathological Gambling Adaptation of Yale-Brown Obsessive Compulsive Scale (PG-YBOCS)

Severity of pathological gambling symptoms was assessed as a secondary outcome with the PG-YBOCS [70]. Internal consistency is high (Cronbach's α = .97). The scale consists of ten questions that measure the severity of gambling symptoms within the past week. The first five questions assess urges and thoughts associated with gambling, whereas the last five questions assess the behavioral component of the disorder. The sum score of each subscale ranges from 0–20. Each subscale can be analyzed separately as well as together as a total score. The total score can be interpreted as follows: 0–7 sub-clinical, 8–15 mild, 16–23 moderate, 24–31 severe and 32–40 extreme gambling symptoms. Originally the questionnaire was used as a semi-structured interview, however, in the present study the PG-YBOCS was administered as an online self-rating questionnaire, which is expected to be unproblematic as both versions (interview and self-rating) show good convergent validity for the YBOCS [71]. In the present study, we used the authorized German translation of the PG-YBOCS by [72].

#### General Anxiety Disorder Screener (GAD-7)

The GAD-7 [73], a short self-report questionnaire, was administered to assess different levels of anxiety over the last two weeks. The questionnaire consists of seven items and can be used as a brief measure for assessing general anxiety disorder (sensitivity 89%, specificity 82% for GAD). It also serves to measure other anxiety disorders, such as panic disorder and social anxiety disorder. The questionnaire has an excellent internal consistency (Cronbach’s alpha = .92). Seven statements are scored from 0–3 resulting in a sum score of 21 with a cut-off score for GAD at 10.

#### Subjective appraisal

Participants in the treatment group were asked for their subjective evaluation and appraisal of the intervention at the post-assessment. Answers could be given on a 3-point Likert-scale ranging from “1 = totally disagree” to “3 = mostly agree”. Moreover, participants were invited to provide direct feedback via a description field. Additionally, they were asked whether they would use the program in the future and whether the program met with their expectations.

### Changes to the study protocol

As the study was part of a larger project, additionally to the reported questionnaires other questionnaires were administered (Gambling Attitudes and Beliefs Survey [GABS], Short Questionnaire on Gambling Behavior (*Kurzfragebogen zum Glücksspielverhalten* [KFG]. As the questionnaires were not relevant for the objective of the present study, they were not included in the analyses. In the initially planned four-arm study, the change in pathological gambling behavior (measured with PG-YBOCS) was intended to be the primary outcome. After the decision was made to split the study into two separate studies, it was decided to choose “change in depressive symptoms” (measured with the PHQ-9) as the primary outcome for the present study as Deprexis is an online program for depression and the aim of the present study (in contrast to the initially planned four-arm study) was to examine its efficacy in treating comorbid depressive symptoms, which makes using a depression as the primary outcome reasonable. Additionally, prior to the start of the study, it was decided to measure depressive symptom severity with the PHQ-9 (instead of the Beck Depression Inventory (BDI-II)). We decided to change the instrument of the primary outcome as the PHQ-9 has several advantages over the BDI-II [74]. Also, questionnaires on suicidality and alcohol dependency were excluded prior to the start of the study for several reasons: Suicidality was assessed with one specific suicide item of the PHQ-9 [75]. As we decided to implement the WSQ, which was originally not planned according to the study protocol, to screen for common psychiatric diseases (including alcohol dependency), an extra questionnaire on alcohol dependency was no longer needed. All changes were decided before the first participant was enrolled in the study. For a detailed overview see **S1 Table. Changes to the study protocol.**

### Strategy of data analysis

To test for baseline differences between the two conditions, independent samples t-tests were used for continuous variables and chi-square tests for categorical variables. Analyses of the primary outcome (PHQ-9) and all secondary outcomes were conducted using analyses of covariance (ANCOVAs). Difference scores (calculated by subtracting pre-treatment scores from post-treatment scores) served as dependent variables and mean baseline scores as covariates. This type of analysis was chosen as it accounts for baseline differences and regression to the mean [76,77]. Intention-to-treat (ITT) and per protocol (PP) analyses were performed. For ITT analyses, all participants who did not meet the criterion for probable problematic gambling in the SOGS screening (only a score of 0 was rated as non-problematic behavior) or did report suicidality at any point of the questionnaire were excluded. Within-group differences were calculated with a paired sample t-test. Missing values for ITT analyses were imputed from main psychopathological measures by means of two methods to address the fact that there is no gold standard procedure to estimate missing values: 1. the expectation-maximizing (EM) algorithm and 2. multiple imputation method [MI; e.g., [78]]. The MI method was performed with the computer software MPlus [Version 7; [79]]. To cope with the high number of missing values, we ran 400 imputations. PP analyses considered all participants with available data of relevant outcome measures in the post-assessment who logged into the Deprexis program at least once. Effect sizes are reported as partial eta squared (η^2^_partial_ ≈ 0.01 small effect, η^2^_partial_ ≈ .06 medium effect, η^2^_partial_ ≈ .14 large effect). All analyses were performed in SPSS version 24. In order to explore for possible subgroup differences, we performed moderation and prediction analyses using the SPSS macro PROCESS (developed by Andrew F. Hayes). We aimed to identify variables that either predict or have an effect on the selective improvement of problem gambling behavior (outcome: PG-YBOCS total difference scores).

### Sample size calculation

The calculation of the optimum sample size was carried out with the software G*Power®. The calculations were made prior to the decision to split the four-arm study into two separate studies, each with two groups. The power calculations for the initially planned ANOVA revealed that for an average effect strength of *f* = . 25 (mean effect), 45 participants would have to be recruited per group for α = . 05 and ß = . 80 (total sample: 180). Our experience with similar studies has shown that a dropout rate of about 20% should be expected. This resulted in a group size of 54 participants for each of the four groups (total sample: 216). However, we decided to include more participants in the study as larger sample sizes are recommended for moderation analyses.

## Results

A total of 7755 subjects accessed the first page of the survey. 286 participants who fulfilled the inclusion criteria completed the entire baseline survey. 141 were randomized to the second sub-study. Of those remaining, we randomized 145 subjects to either the Deprexis or the control group (Fig 1). For analyses, we had to exclude four participants because of a SOGS total score = 0 and one participant because of suicidality according to WSQ. Our final intention-to-treat sample consisted of 140 subjects; 71 were randomized to the Deprexis group and 69 to the wait-list control group.

### Baseline characteristics and attrition

The baseline characteristics of the sample are presented in Table 1. 76.4% of participants were male, on average 35.71 (*SD* = 10.21) years old with 10.70 (*SD* = 1.50) years of formal school education. Most participants (87.1%) were of German origin, were living with their partner with or without children (49.3%) and 31.4% lived alone. The majority (68.6%) were full-time employed, 12.1% were unemployed.

**Table 1:** Baseline characteristics. Percentage, means and standard deviations (in brackets).

	Deprexis (*n* = 71)		Wait-list (*n* = 69)	
	*N* (%)	*M* (*SD*)	*N* (%)	*M* (*SD*)
**Gender (male)**	76.06		76.81	
**Age in years**		34.42 (10.74)		37.04 (9.53)
**Education in years**		10.72 (1.48)		10.68 (1.54)
**Nationality (German)**	90.15		84.06	
**Currently in psychotherapy**	5.63		21.73	
**Psychotropic medication**	2.82		7.25	
**Self-help**	7.04		11.59	
**Age at first game**		20.61 (9.25)		20.43 (7.91)
**Age at frequent gambling**		23.46 (9.50)		24.09 (8.86)
**Currently in suspension**	16.9		10.1	
**PHQ-9**		10.80 (5.81)		11.02 (5.37)
**GAD-7**		8.76 (5.31)		8.94 (4.95)
**SOGS total score**		9.75 (3.14)		9.71 (3.24)
**PG-Y-BOCS total score**		17.92 (6.70)		17.68 (6.91)
**PG-Y-BOCS behavior**		8.94 (3.46)		8.68 (3.87)
**PG-Y-BOCS thoughts**		8.97 (3.59)		9.00 (3.55)

For the total sample (*n* = 140), 13.6% were currently using professional psychotherapeutic help, 5% were taking psychotropic medication and 9.3% were already using some other form of self-help (e.g. self-help groups, internet-forums) at baseline. In the control group, significantly more participants were currently in psychotherapeutic treatment (21.7%) in comparison to the intervention group (5.6%).

Overall, the majority of participants (62.1%) had their first gambling experience by the age of 18 or before. 40.7% reported that they started frequent gambling between the ages of 19 and 29 years. Most participants were neither in the past nor at the present suspended from gaming (79.3%). 56.43% reported monthly losses of 500 to 1500 euros, 21.4% reported no debts at baseline, 19.2% reported debts between 1,100 and 1,700 euros and 17.14% debts over 10,000 euros.

According to the WSQ, 45% of subjects were screened positive for OCD, 44.3% for Specific Phobia, 41.4% for PTSD, 32.2% for Social Phobia, 31.4% for Generalized Anxiety Disorder, 34.3% for Panic Disorder, 20% for Depression, 12.1% for Agoraphobia as well as for Alcohol Abuse / Dependence, and 7.14% for Panic with Agoraphobia. We would like to point out that the WSQ is regarded as a valid screening tool, however it cannot replace an elaborate diagnostic interview. A positive WSQ screening result requires further diagnostic procedures to verify diagnoses. Additionally, specificity for PTSD (0.52) and Special Phobia (0.73) is rather moderate, which may lead to an overestimation of those diagnoses [80].

Depression symptom severity according to PHQ-9 was moderate, on average (*M* = 10.91, *SD* = 5.58); average anxiety severity measured by GAD-7 was mild (*M* = 8.85, *SD* = 5.12).

According to SOGS, 3.6% had at least some problems with gambling, whereas 96.4% were screened as probable pathological gamblers (*M* = 9.73, *SD* = 3.18). Overall gambling-related symptom severity, measured by PG-YBOCS, was moderate (*M* = 17.80, *SD* = 6.78). The internal consistencies (Cronbach’s α) of the main outcomes were determined for the whole sample (*n* = 140) and were satisfactory to good (Y-BOCS total: α = .851, PHQ-9: α = .853, GAD-7: α = .884, SOGS: α = .741).

### Completion rate

The final sample consisted of 140 participants. Of those, 62 (44.3%) completed the post assessment. Completion rate was significantly higher in the wait-list control group (56.5%), compared to the intervention group (32.4%), χ(1) = 8.26, *p* = .004. Across the whole sample, completers and non-completers did not significantly differ as to gender, years of education, the frequency of current psychotherapy and psychotropic medication. However, completers were significantly older (*M* = 37.89, *SD* = 9.89) compared to the non-completers (*M* = 33.99, *SD* = 10.20), *F*(1; 138) = 2.28, *p* = .024. Regarding the psychopathology measures at baseline, non-completers had higher scores indicating more severe symptoms on the GAD-7, PG-Y-BOCS behavior subscale and the PG-YBOCS total scale than completers (all *p*s < .02).

### Intention-to-treat analyses

For the ITT analyses, the expectation-maximization (EM) method as well as multiple imputations (MI) were used to estimate missing values. Results of the ITT analyses using EM are summarized in Table 2, indicating significant group differences in favor of the intervention group compared to the wait-list group regarding all parameters except the PG-YBOCS behavior scale. For the reduction of depressive symptoms, as measured with the PHQ-9, group differences showed a strong significant effect for Deprexis relative to the control condition, *F(*1;137) = 19.64, *p* < .001, ηp2 = .125. This result remained unchanged when missing data were replaced using MI (*p* = .030), but effects were not significant for any other measures.

**Table 2:** Between group differences across time. Means and standard deviations of PP-cases [within-group differences are in square brackets].

	Wait-list (*n* = 39)			Deprexis (*n* = 23)			Between-group difference pre-post; ANCOVAs with baseline scores as covariates	
	Pre *M* (*SD*)	Post *M* (*SD*)	Mean difference (95% CI)	Pre *M* (*SD*)	Post *M* (*SD*)	Mean difference (95% CI)	Per Protocol (PP)	Intention to treat (ITT)*
**Primary outcome**								
**PHQ-9**	10.26 (5.11)	8.26 (5.14) [*]	2.00 (.372–3.628)	9.39 (4.46)	5.74 (4.52) [***]	3.65 (1.742–5.562)	*F* (1;59) = 3.40, *p* = .070, ηp2 = .054	*F*(1;137) = 19.64, *p* < .001, ηp2 = .125; [*p* = .030]
**Secondary outcomes**								
**SOGS**	9.11 (2.98)	6.53 (4.08) [****]	3.09 (1.758–4.416)	8.43 (2.33)	5.35 (3.77) [****]	3.09 (1.758–4.416)	*F* (1;58) = .55, *p* = .462, ηp2 = .009	*F*(1;137) = 8.26, *p* = .005, ηp2 = .057; [*p* = .118]
**PG-YBOCS** **total**	16.13 (7.36)	13.51 (8.83) [*]	5.30 (2.052–8.557)	16.52 (6.03)	11.22 (7.67) [***]	5.30 (2.051–8.557)	*F* (1;59) = 1.76, *p* = .190, ηp2 = .029	*F*(1;137) = 4.01, *p* = .047, ηp2 = .028; [*p* = .295]
**PG-YBOCS** **behaviour**	7.56 (4.19)	6.33 (4.74)	1.23 (-.187–2.649)	8.26 (3.31)	5.30 (4.25) [**]	2.95 (.977–4.936)	*F* (1;59) = 1.68, *p* = .200, ηp2 = .028	*F*(1;137) = 3.21, *p* = .076, ηp2 = .023; [*p* = .361]
**PG-YBOCS** **thoughts**	8.56 (3.55)	7.18 (4.24) [*]	1.38 (.184–2.585)	8.97 (3.59)	5.91 (3.86) [**]	2.35 (.453–4.069)	*F* (1;59) = 1.34, *p* = .252, ηp2 = .022	*F*(1;137) = 3.99, *p* = .048, ηp2 = .028; [*p* = .489]
**GAD-7**	7.51 (4.64)	7.23 (4.90)	.28 (-1.205–1.769)	7.91 (5.01)	5.65 (4.20) [*]	2.26 (.453–4.069)	*F* (1;59) = 3.06, *p* = .085, ηp2 = .049	*F*(1;137) = 13.10, *p* < .001, ηp2 = .087; [*p* = .053]

We present pairwise data of PP-analyses. For this reason pre-scores may deviate from those reported in Table 1.* ITT analyses were computed with EM as method for missing values.

[*] = p ≤ .05

[**] = p ≤ .01

[***] = p ≤ .005

[****] = p ≤ .001

### Per protocol analyses

A total of 39 patients in the wait-list group and 23 patients in the Deprexis completed the trial according to protocol. Results are summarized in Table 2. For the primary outcome (PHQ-9) as well as all secondary outcomes, the PP-analyses revealed no significant difference between the two groups. For within-group differences, subsidiary paired sample t-tests showed a significant reduction of all variables from baseline to post-assessment for the intervention group. Especially for the PHQ-9, the SOGS and the PG-YBOCS thoughts subscale, the pre to post differences were highly significant. For the wait-list group, within group differences over time were also significant for most scales but at lower magnitudes.

### Subjective appraisal and usage

The subjective appraisal of the intervention is summarized in Table 3. In general, the intervention was evaluated positively. Almost all participants (95.3%) considered the program suitable for self-application and found the contents comprehensible (100%). The clear majority thought that the program was generally useful (81%), was able to use the program regularly and considered the program as a useful adjunct to psychotherapy. Nonetheless, only 42.9% thought that their gambling problem reduced by using the program and a high proportion (80.9%) of the participants said they had to push themselves to use the program. No adverse events were noted.

**Table 3:** Subjective appraisal of Deprexis (*n* = 21).

Items	*M*	% positive	*SD* (Range)
**1. I think the program is suitable for self-application.**	2.48	95.3	.60 (1–3)
**2. My gambling problem reduced by using the program.**	1.57	42.9	.75 (1–3)
**3. I think the instructions were written comprehensibly.**	2.86	100	.36 (1–3)
**4. I think the program was useful.**	2.19	81.0	.75 (1–3)
**5. I was able to use the program regularly over the past weeks.**	1.86	71.4	.66 (1–3)
**6. I had to push myself to use the program.**	2.38	80.9	.81 (1–3)
**7. I consider the program to be a useful adjunct to psychotherapy.**	2.24	76.2	.83 (1–3)
**8. The program is not relevant for my gambling-related symptoms.**	2.24	71.4	.89 (1–3)

The time that participants used Deprexis was also recorded. The intervention was used for an average of *M* = 82.60 minutes (*SD* = 53.87, range = 5.00–185.00).

### Moderator and predictor analyses

We conducted moderation and prediction analyses with the per protocol data (*n* = 62). Table 4 provides data for moderator effects on the improvement of problem gambling behavior over time (dependent variable: PG-YBOCS total difference scores; independent variable: group allocation). The results were assessed with the SPSS macro PROCESS by Hayes. All parameters were set to the standard mode. PROCESS reports both main effects (predictors) and interaction effects (moderators). In addition, we report upper and lower limits of bootstrapping confidence intervals based on 5000 samples. Since the response scales of the moderator variables (nominal or metric, descending or ascending) are different in each case, it is not sufficient to look only at the value (positive or negative) of the regression coefficient. For an adequate interpretation, one has to look at the response scale of the moderator variable and the beta coefficient. The results show that participants in the intervention group benefited more in terms of their problem gambling symptoms when they have a low vocational status, report a loss of interest/joy (PHQ item 1), do not (or rarely) play dice games, do not (or rarely) play at casinos, whose partners do not have gambling problems and who did not borrow money from other family members/relatives, as well as those who do not report having panic symptoms. Moreover, patients with higher baseline feelings of loneliness had significantly less severe gambling-related symptoms following the intervention. At trend level, a low level of education (fewer years in school), high depressive symptoms (e.g., loss of interest/joy, poor opinion of oneself, no slowing of movements or agitation over the last week, loss of interest and pleasure (WSQ item 2)) predicted better outcome for Deprexis.

**Table 4:** Moderators for PG improvement (PG-YBOCS total difference scores, means are centered).

Outcome Parameter	*B*	*SE*	*t*	*p*	LLCI	ULCI	*p* for -1SD	*p* for 0	*p* for +1SD
**Number of years of education**	.802	.455	1.763	.083	-.109	1.712	.028	.178	.723
**Vocational status**	.709	.312	2.273	.027	.085	1.334	.025	.168	.549
**PHQ Item 1 (loss of interest/joy in activities)**	-1.718	.791	-2.171	.034	-3.303	-.134	.533	.172	.018
**PHQ Item 6 (Feeling bad about yourself/ to be a loser)**	-1.359	.749	-1.814	.075	-2.859	.141	.888	.108	.025
**PHQ Item 8 (Moving or speaking slowly/ agitation)**	2.476	1.243	1.991	.051	-.013	4.967	.030	.399	.347
**WSQ Item 2 (depression)** *	2.557	1.405	1.820	.074	-.256	5.370	.029	-	.971
**WSQ Item 4 (panic disorder)**	1.756	.671	2.619	.011	.414	3.098	.016	.170	.404
**SOGS Item 1.4 (dice games)**	7.411	2.922	2.537	.014	1.563	13.259	.056	-	.036
**SOGS Item 1.5 (casino visit)**	1.922	.771	2.493	.016	.379	3.466	.016	.237	.363
**SOGS Item 3.5 (partner)**	6.972	3.109	2.243	.029	.750	13.195	.058	-	.067
**SOGS Item 14 (gambling debts)**	3.648	1.500	2.432	.018	.645	6.651	.007	-	.940
**SOGS Item 16.3 (borrowed money from family members/ relatives)** *	1.946	.929	2.096	.041	.087	3.805	.018	.151	.931
**Motivation Item 12 (feeling lonely)** *	4.022	1.365	2.946	.005	1.289	6.754	.006	-	.113

*B* = beta coefficient; *SE* = standard error LLCI = lower limit confidence interval; ULCI = upper limit confidence interval. The last three columns give the p-values when values are one standard deviation above or below or are equal to the mean.

*Response scale of the items is descending from severe to not present (e.g., 1 = depression, 2 = no depression).

Table 5 shows the predictors of improvement in problem gambling behavior (PG-YBOCS), irrespective of group allocation. As in Table 4, it is not sufficient to look only at the value of the regression coefficient (positive or negative) for interpreting the results as the items were rated in different directions (sometimes a low value indicates high symptom endorsement, and sometimes it indicates the opposite). The results show that several items of the SOGS as well as the PG-YBOCS and also PG-YBOCS total scores at baseline predicted overall improvement: severe symptoms at baseline were associated with greater improvement at post suggesting regression to the mean. We would like to point out that some items within both sets of analyses (e.g., moderation and prediction) did not reach statistical significance (< .05) but trend level (< .1). As noted, we conducted explorative analyses and therefore decided to also report models with statistical trend.

**Table 5:** Predictors for PG improvement (PG-YBOCS total difference scores, means are centered).

Outcome Parameter	*B*	*SE*	*t*	*p*	LLCI	ULCI
**SOGS Item 10 (Wanted to stop gambling but thought could not)** *	-7.055	3.318	-2.127	.038	-13.696	-.415
**SOGS Item 16.2 (Borrowed money from partner for gambling)** *	2.688	1.313	2.047	.045	.060	5.316
**Motivation Item 11 (When money is directly available)** *	4.774	2.187	2.183	.033	.397	9.151
**PG-YBOCS Item 1 (Time occupied by gambling thoughts/urges)**	1.805	.939	1.923	.060	-.074	3.685
**PG-YBOCS Item 2 (Interference due to gambling thoughts/urges)**	1.874	1.009	1.856	.069	-.147	3.894
**PG-YBOCS Item 5 (Degree of control over gambling thoughts/urges)**	2.485	1.132	2.195	.032	.218	4.752
**PG-YBOCS Item 6 (Time occupied by gambling behavior)**	1.907	.877	2.175	.034	.152	3.662
**PG-YBOCS Item 7 (Interference due to gambling behavior)**	3.353	.830	4.043	< .001	1.693	5.014
**PG-YBOCS Item 10 (Degree of control over gambling behavior)**	2.725	.835	3.262	.002	1.053	4.397
**PG-YBOCS Thoughts scale**	.596	.295	2.020	.048	.006	1.186
**PG-YBOCS Behavior scale**	.779	.246	3.167	.003	.287	1.271
**PG-YBOCS Total scale**	.385	.140	2.752	.008	.105	.666

*B* = beta coefficient; *SE* = standard error; LLCI = lower limit confidence interval; ULCI = upper limit confidence interval

*Response scale of the items is descending from severe to not present (e.g., 1 = loss of control, 2 = no loss of control).

## Discussion

Gambling disorder is a disorder related to both addiction (DSM-5) and impulse control disorders (ICD-10). It is a frequent but hidden disorder; only a minority of patients seeks treatment and dropout rates are high for those who initiate a therapy [13,81]. Therefore, alternative and especially low-threshold treatment forms, such as internet-based treatment, could help bridge the treatment gap. Advantages of internet interventions are their anonymity, their accessibility even in remote areas, and their low entry threshold for sufferers with incomplete insight into their disorder. A large proportion of individuals suffering from gambling problems have comorbid affective symptoms, and although the precise relationship between gambling and emotion disorders is still not fully understood, depressive symptoms play an important role in the emergence and maintenance of gambling problems, according to most experts [7,17,21]. For most individuals with problematic gambling, the gambling itself plays a central and structuring role in life. In many cases, gambling becomes the most important activity and even outranks family, friends and hobbies [82]. The function of the disorder thus plays a major role in treatment motivation and compliance. For this purpose, our intention was to work on both the causal antecedents and “side-effects” of the gambling problem (e.g., reduce depressive symptoms, strengthen self-esteem, encourage positive activities).

The present study therefore adopted a low-threshold online approach targeted at depressive symptoms, as they often precede to pathological gambling [21]. Pathological gambling may represent a dysfunctional coping strategy to deal with affective and social symptoms, such as loneliness and sadness ("problem gamblers"/"escape gamblers"). In addition, negative affect including depression are frequent triggers for problem gambling, which in turn may lead to secondary depression because of debt and guilt [7]. We thus aimed to "kill two birds with one stone" and hypothesized that a reduction in depressive symptoms could also lead to a decline in gambling problems and vice versa.

### Summary of main results

As hypothesized, the online-intervention Deprexis led to a significant decrease of both depressive symptoms and problem gambling behavior according to the ITT analyses using EM with moderate to strong effect sizes. The PP analyses failed to yield significant results due to high rates of non-completion and resulting low statistical power; ITT analyses using multiple imputation suggested a steeper decline of depression in the Deprexis than the control group. Yet, the results for other parameter effects were non-significant.

Many studies with internet-based treatment are plagued by high dropout rates, particularly in those without financial reimbursement like ours. One study [30] found that being male, having a lower educational level, younger age and comorbid anxiety symptoms are significantly related to dropout in online studies. In our sample we had a high proportion of individuals with these characteristics (e.g., more than 75% male participants). However, contrary to previous findings we could not find any significant relationship between gender and completion rate. In this context, the ambivalent treatment motivation of problematic gamblers needs to be considered as well. A study on dropout in pathological gamblers [81] reports that a high proportion of treatment seekers do not even start the treatment. It appears that many individuals with problematic gambling behavior are not fully determined to quit gambling. Instead, they often aim to get rid of problems that appear as a consequence of the problematic gambling.

We found that several characteristics play a moderating role in the course of treatment. Individuals with higher depressive symptomatology and those who gamble as a reaction to feelings of loneliness seem to profit more from the intervention in terms of an improvement of gambling behavior. Furthermore, having severe gambling-related symptoms at baseline was a predictor for problem gambling improvement following the intervention. We would like to point out that some effects of the moderation and prediction analyses were only significant at trend level, so those findings need to be interpreted with caution. As the overall gambling related symptom severity measured by PG-Y-BOCS was moderate in our sample, we hypothesize that the effect of the intervention might be larger in a sample of individuals with more severe symptoms. The fact that the wait-list group also showed an improvement in symptoms can also be discussed under this point. The literature indicates that problematic gamblers have less stable symptoms than pathological gamblers. Our sample is composed of participants who have the subjective feeling of having a gambling problem and therefore more participants are represented with moderate symptoms who have not yet reached the level of pathological gambling. We assume that with other inclusion criteria (pathological gamblers only), the decline in the wait-list group would be less and the intervention would therefore show greater effect sizes.

### Limitations

Before we turn to the conclusions of our study, we need to acknowledge a number of limitations. First, completion rates were better than in other internet trials on problematic gambling [51] but non-completion was substantial, which compromises our ability to draw firm conclusions. The dropout rate was significantly higher in the intervention group, which might reflect the fact that the intervention did not directly meet the participants’ expectations/needs, particularly gambling problems. One may also argue that participants in the control group had more to gain from the re-assessment, as they received access to the program upon completion. In view of many studies that the efficacy of Deprexis with other samples [for a meta analysis see [42]], it may be argued that the high dropout rate in this study is unlikely due to a general problem with the intervention. Indeed, in some studies with Deprexis [33] dropout rates as low as 0% have been observed at posttreatment (in a clinician-guided treatment arm). The high non-completion rates we observed suggest, if anything, that adapting the intervention to better match the needs of people with gambling disorder might improve adherence. We will turn to this point below. To date, there are no studies that report findings on predictors for treatment adherence in internet interventions for problem gamblers, however, there are studies on predictors for adherence in samples with a different psychopathology. Contrary to the findings of an internet intervention study on the predictors for adherence in depression [83], our analyses showed that non-completers were significantly younger and had more severe anxiety and gambling-related symptoms at baseline. More affected individuals might have had a more ambiguous motivation to stop gambling or were too stressed to concentrate on the more demanding texts of Deprexis. Additionally, it could also be hypothesized that more severely impaired individuals felt less "understood" by Deprexis, as it is not targeted at problematic gambling. The fact that non-completion was higher in the intervention group as well as in participants with more severe symptomatology at baseline also highlights the fact that the results of the ITT-analyses need to be interpreted cautiously. Both statistical methods we used for dealing with missing cases assume that missing data occurs randomly. However, the differences with regard to completers and non-completers indicate that the data is not entirely missing at random.

Secondly, studies with larger samples and longer follow-up intervals are needed to improve power and to assess whether effects are sustained. It would be interesting to see whether the intervention has long-term effects, although we would then expect an even higher dropout rate at follow-up. Lower attrition rates at follow-up assessments might be achieved by providing an incentive such as vouchers, alternative therapeutic material or small amounts of money. Some studies have suggested that this intervention does facilitate long-term improvements [84].

Thirdly, the program was not adapted to the specific needs and problems of the target population, so that the potential of online interventions in this group might have been underestimated. This might also be one reason for the high dropout rate as well as the negative results on some items of the subjective evaluation of the program. Although the overall evaluation was clearly positive, the majority of the participants considered the program not suitable for their problems and did not think that using the program reduced their gambling problems. This feedback has important implications for the development of programs for individuals with problem gambling in the future. It is hypothesized that an adaptation of the program to gambling-specific topics may result in greater acceptance and adherence, which in turn might improve treatment outcome.

Fourthly, the population was self-selected and might not have been representative of all problem gamblers. People with higher education were over-represented. This is noteworthy as we found that participants with a lower educational background could profit more from the intervention. People with a higher education might have adopted/knew a number of coping strategies conveyed in the training before and thus gained less new knowledge/insights compared to individuals with poorer resources. As the study was conducted in German language, we were unable to reach migrants with insufficient language skills, which form a large subgroup of problem gamblers in Germany.

Also, due to the controversy regarding the subdivision of problematic and pathological gamblers, our analyses did not differentiate between these two groups but included all participants with self-reported gambling problems and a SOGS total score > 0. Expanding the population of pathological gamblers by including those who probably have less severe symptoms could result in a higher Type I error.

Lastly, within the study all gathered data is based on self-reports, which might differ from external assessments. Future studies should therefore consider expert ratings via telephone in order to verify responses as well as diagnoses. This may however ward off some individuals as some studies show that guided interventions are at risk of higher non-completion rates than unguided ones.

### Implications

For future studies, it is recommended to either develop programs tailored to the symptoms and needs of gamblers or to adjust existing programs; that is, to supplement available programs with modules dealing, for example, with money management, coping with the urge to gamble and relapse prevention. Furthermore, gambling-specific cognitive biases such as "illusion of control" and "gambler's fallacy" are an important treatment target as they appear to be one of the most determining factors in the emergence and maintenance of the disorder [85,86]. Smartphone-apps are deemed especially powerful to transpose contents into daily life and sustain improvements. It might be wise to omit formulations that contain the terms "depression" or "depressive symptoms" and instead address emotional problems in a subtle way. As many problem gamblers do not seek professional help because they do not think that they have a psychiatric problem at all, it might help to not label the users as pathologic. The program may also teach techniques to regulate impulse control. In the long run, translations of the program are needed that should consider cultural differences, as many people with problematic gambling behavior have a migration background.

## Conclusion

To the best of our knowledge, ours is the first study to investigate online-based treatment for depressive symptoms in problematic gamblers. We found promising results that support the use of internet-based treatment options for individuals with problematic or pathologic gambling behavior. However, our study faced several limitations and the results need to be replicated by independent investigators, preferably with more gambling-specific programs.

To conclude, the present study suggests that an internet intervention program not directly targeting problematic gambling but one of its possible psychological underpinnings, depression, may lead to a decline in depression as well as gambling-related symptoms (at least for one of the ITT analyses); this effect is likely augmented if the program is tailored to the specific needs of this heterogeneous population. Nonetheless, the results need to be interpreted cautiously. However, based on the robust evidence from previous studies regarding the effectiveness of internet-based therapies for substance use disorders as well as depression (27,36,42,43), our results suggest that this alternative, anonymous low-threshold treatment option could also serve as a meaningful add-on or facilitator to conventional therapy in individuals with problematic gambling behavior or for those who are not (yet) willing to have therapeutic face-to-face contact.

### Conflict of interest statement

The study was funded by the Gauselmann AG, a German gaming and gambling company. The Gauselmann AG had no involvement in the study design, implementation, data collection and analyses, the manuscript, or the submission process. The declaration of the German Interstate Gambling Treaty claims that the gambling industry is required to spend money on the prevention of pathological gambling.

## Supporting information

S1 CONSORT Checklist (DOC)

S1 Table Changes to the study protocol. (DOCX)

S1 Data Set (SAV)

S1 Trial Protocol Within ethics application (in German). (PDF)

S1 Translation Of relevant parts of trial protocol. (DOCX)
