# Correction: Effects of a depression-focused internet intervention in slot machine gamblers: A randomized controlled trial

**DOI:** 10.1371/journal.pone.0203145

**Published:** 2018-08-23

**Authors:** Lara Bücker, Julia Bierbrodt, Iver Hand, Charlotte Wittekind, Steffen Moritz

There are errors in the first paragraph of the "Treatment allocation" section of the Methods and Materials. The allocation aspects are not clear. The correct paragraph is: This study was part of a larger project investigating the effectiveness of an internet intervention (Deprexis) and an online training program (retraining) in problem gamblers. Initially, a four-arm study was planned. Before the start of the recruitment, we decided to split the four-arm study into two separate studies, each with one intervention and one control group. This was done because the two interventions pursue different goals and therefore necessitate different primary and secondary outcomes. Data on the other study, which evaluated a training program (retraining) by means of the Approach-Avoidance Task [60] will be presented in a separate paper (Wittekind et al., submitted). Participants who completed the baseline survey were randomly allocated to either Deprexis or the no intervention control group in a pseudo-random order (based on the date and time of the baseline assessment). The randomization sequence was generated with the computer software Research Randomizer [61]. The random allocation rule was 1:1:1:1, participants were evenly randomized across conditions. Enrollment was self-selected (via online registration) and participants were then allocated to conditions according to a computer-generated randomization plan (participants were randomized according to the time of registration). This procedure is best described as centralized assignment. As a consequence, the risk of bias is low as there was no further information about the participants available. Due to the online setup of the study, concealed allocation was different from the standard case where enrollment is performed by team members, thus, no special efforts were deemed necessary for concealment. After finishing the baseline assessment, the experimental group received an e-mail with a registration code for the Deprexis program, whereas the control group received the information that they will get access to the program upon completion of the post-assessment after eight weeks. All participants were informed that they could contact the study team for questions at any time and were allowed to use any form of treatment during the study period, including medication and psychotherapy. The first participant was enrolled in the study on April 25^th^ 2014, the last participant enrolled completed the post survey on August 23^rd^ 2016. Fig 1 shows a flowchart of the study selection.

## References

[1] Bücker L, Bierbrodt J, Hand I, Wittekind C, Moritz S (2018) Effects of a depression-focused internet intervention in slot machine gamblers: A randomized controlled trial. PLoS ONE 13(6): e0198859 10.1371/journal.pone.0198859 29883479 PMC5993308

