# Supplementary material for: Effects of a depression-focused internet intervention in slot machine gamblers: A randomized controlled trial
Source: PLoS One. 2018 Jun 8;13(6):e0198859. doi: 10.1371/journal.pone.0198859 (PMC5993308; doi:10.1371/journal.pone.0198859)
Supplement: S1 Table — (DOCX) [file pone.0198859.s002.docx]

**S1 Table.** Changes to the Study Protocol

| **Domain** | **Original Study  Protocol** | **Implemented Change** | **Rationale/Comments** |
| --- | --- | --- | --- |
| **Study design (study arms)** | Initially, a randomized controlled trial with four study arms was planned. The main objective of the study was to investigate to what extent two experimental study arms (Deprexis [1] and retraining [2]) lead to a differentially greater reduction in pathological gambling than an active control intervention [3] or a wait-list control condition [4]. | Prior to the start of recruitment, we decided to split the study into two separate studies for each of the two conditions as the interventions target different outcomes. The present article deals with the Deprexis [1] and the wait-list control conditions [4]. Data on the other study will be presented elsewhere. | The two interventions (Deprexis and retraining) pursue different aims and therefore different outcomes are expected. For Deprexis, a reduction in depression (primary outcome) was expected to have an effect on gambling (secondary outcome). Retraining targets gambling directly; no effect on depression was expected. |
| **Primary outcome** | In the initially planned four-arm study, the change in pathological gambling behavior (measured with PG-YBOCS) was intended to be the primary outcome. | After the decision was made to split the study, it was decided to choose “change in depressive symptoms” (measured with the PHQ-9) as the primary outcome for the present study. | Deprexis is an online program for depression, and the primary aim of the present study (in contrast to the initially planned four-arm study) was to examine the efficacy of the internet intervention Deprexis in treating comorbid depressive symptoms in problematic and pathological slot-machine gamblers, which makes using a depression measure as the primary outcome measure reasonable. |
| **Primary outcome measure** | Depressive symptom severity should originally be measured with the BDI-II. | Prior to the start of the study, it was decided to measure depressive symptom severity with the PHQ-9 (instead of the BDI-II). The BDI-II was therefore not administered. | We decided to change the instrument of the primary outcome as the PHQ-9 has several advantages over the BDI-II. It consists of fewer items (9 vs. 21) and the items are based on diagnostic criteria for depression (DSM), while psychometric properties do not substantially differ between both scales (1). |
| **Secondary outcomes** | Administration of the following questionnaires was initially planned:   - Beck-Depression Inventory (BDI-II) - Suicide Behaviors Questionnaire-Revised (SBQ-R) - Generalized Anxiety Disorder 7-Item (GAD-7) - Alcohol Use Disorders Identification Test (AUDIT) - South Oaks Gambling Scale (SOGS) - Gambling Attitudes and Beliefs Scale (GABS) - Short Questionnaire on Gambling Behavior *(Kurzfragebogen zum* *Glücksspielverhalten* (KFG)) - Pathological Gambling Y-BOCS (PG-YBOCS) | Administration of the following questionnaires was performed:   - Patient Health Questionnaire 9 items depression module (PHQ-9) - Generalized Anxiety Disorder 7-Item (GAD-7) - South Oaks Gambling Scale (SOGS) - Gambling Attitudes and Beliefs Scale (GABS) - Short Questionnaire on Gambling Behavior *(Kurzfragebogen zum Glücksspielverhalten* (KFG)) - Pathological Gambling Y-BOCS (PG-YBOCS) - Web Screening Questionnaire (WSQ) | 1. The BDI-II, the SBQ-R and the AUDIT were excluded prior to the start of the study for several reasons (see above for the BDI-II): Suicidality was assessed with one specific suicide item of the PHQ-9, so the SBQ-R was no longer needed and thus excluded (2). As we decided to implement the WSQ to screen for common psychiatric diseases (including alcohol dependence, the AUDIT was no longer needed. 2. As the study was part of a larger project, in addition to the reported questionnaires other questionnaires were administered (GABS and KFG). As those questionnaires were not relevant for the objective of the present study, they were not included in the analyses of the present study. |
| **Sample size** | The power analysis using G*Power indicated that a sample size of 54 participants for each group would be adequately powered (for a medium effect size *f =* .25, α = .05, ß = .80 and a dropout rate of 20%). | The final sample size was 140. | We decided to include more participants in the study than initially planned as larger sample sizes are recommended for moderation analyses. |

References

1. Titov N, Dear BF, Mcmillan D, Anderson T, Zou J, Sunderland M. Psychometric comparison of the PHQ-9 and BDI- II for measuring response during treatment of depression. Cogn Behav Ther. 2011;40(10):126-136.

2. Uebelacker LA, German NM, Gaudiano BA, Miller IW. Patient health questionnaire depression scale as a suicide screening instrument in depressed primary care patients: a cross-sectional study. Prim care companion CNS Disord. 2011;13(1).
