# Supplementary material for: Effects of a depression-focused internet intervention in slot machine gamblers: A randomized controlled trial
Source: PLoS One. 2018 Jun 8;13(6):e0198859. doi: 10.1371/journal.pone.0198859 (PMC5993308; doi:10.1371/journal.pone.0198859)
Supplement: S1 Trial Protocol — (PDF) [file pone.0198859.s004.pdf]

Antrag auf Stellungnahme der Ethikkommission der DGPs

1. Bezeichnung des Forschungsvorhabens
„Computerbasiertes Trainingsprogramm für Menschen mit Glücksspielproblemen“

2. Name und Kontaktdaten des Antragstellers (Dienstanschrift):
Prof. Dr. Steffen Moritz
Universitätsklinikum Hamburg-Eppendorf
Klinik und Poliklinik für Psychiatrie und Psychotherapie
Arbeitsgruppe Klinische Neuropsychologie
Martinistraße 52
20246 Hamburg
moritz@uke.uni-hamburg.de
Telefon: (040) 7410-56565
Telefax: (040) 7410-57566

3. Angaben zu den Rahmenbedingungen des Vorhabens
Es handelt sich um ein durch die Automatenindustrie bewilligtes Projekt. Eine Stellungnahme der Ethikkommission wird angeraten.

4. Gegenstand und Verfahren des Vorhabens
4.1 Gegenstand
Bei der Studie handelt es sich um die Untersuchung der Effektivität von computerbasierten Trainingsprogrammen bei Betroffenen mit pathologischem Glücksspiel. Bereits bestehende oder parallel durchgeführte Therapien bleiben durch die Studie unangetastet. Das Hauptziel der Studie ist, zu untersuchen, inwieweit zwei experimentelle Studienarme (Deprexis und Retraining) zu einer differentiell stärkeren Reduktion des pathologischen Glücksspiels führen als eine aktive Kontrollintervention oder eine Wartelistenkontrollbedingung. Sowohl das Programm „Deprexis“ als auch das „Retraining“ stellen bereits auf ihre Wirksamkeit hin überprüfte Selbsthilfeprogramme dar („Deprexis“ zur Reduktion von depressiven Symptomen; Retraining zur Reduktion von Abhängigkeitserkrankungen) und wurden im Rahmen früherer Studien von der Ethikkommission der Deutschen Gesellschaft für Psychologie (DGPs) als unbedenklich eingestuft. Es wird erwartet, dass die beiden Interventionsprogramme zu einer signifikanten Reduktion des pathologischen Spielverhaltens (gemessen über die PG Y-BOCS) führen im Vergleich zur aktiven Kontroll- und Wartekontrollbedingung mit deutlichem Vorteil für die „Ursachentherapie“ (deprexis). Analog Hand (2004) wird erwartet, dass „Retraining“, welches als Symptom-Verfahren beschrieben werden kann, besonders bei „positiven Verstärkern“ (v.a. Sucht nach Unterhaltung/Abwechslung; *action-seeker*) wirksam ist, während „Deprexis“ eher Effektivität entfaltet bei sog. „negativen Verstärkern“ (Betroffenen mit primären psychischen (z.B. affektiven) Störungen; *escape-seeker*).

4.2 Methoden
Bei der Studie handelt es sich um eine vierarmige randomisiert-kontrollierte online-Interventionsstudie: 1. Deprexis *(Behandlung primärer Depression)*, 2. Retraining *(Behandlung des pathologischen Spielverhaltens)*, 3. aktive Kontrollbedingung und 4. Wartekontrollgruppe. Es sind zwei Untersuchungszeitpunkte vorgesehen, wobei der Interventionszeitraum (prä-post) acht Wochen beträgt. Zu beiden Messzeitpunkten werden soziodemografische und psychopathologische Daten in einer Internetbefragung über Unipark® gewonnen. In Tabelle 1 sind die Instrumente dargelegt, die zu den beiden Erhebungszeitpunkten vorgesehen sind.
Am Ende der Studie erhalten alle Teilnehmer zur Förderung der Studienadhärenz Selbsthilfemanuale unserer Arbeitsgruppe. Die Teilnahme an der beschriebenen Studie wird auch ermöglicht, wenn bereits andere Therapien (z.B. Psychopharmako- oder Psychotherapie) laufen.

Tabelle 1. Erhebungsinstrumente über die zwei Untersuchungszeitpunkte

Instrument	Baseline	Post (8 Wochen)
Soziodemographischer Fragebogen	X	X
Kurzanamnese	X	
Allgemeine Psychopathologie		
Beck-Depressions Inventar (BDI-II)	X	X
Suicide Behaviors Questionnaire-Revised (SBQ-R)	X	X
Generalized Anxiety Disorder 7-Item (GAD-7)	X	X
Alcohol Use Disorders Identification Test	X	X

Instrument	Baseline	Post (8 Wochen)
(AUDIT)		
Fragebögen zu verschiedenen Aspekten pathologischen Glücksspiels		
Fragen zu Spielertypen	X	
Fragen zum Glücksspielverhalten	X	
South Oaks Gamling Scale (SOGS)	X	X
Gambling Attitudes and Beliefs Scale (GABS)	X	X
Kurzfragebogen zum Glücksspielverhalten (KFG)	X	X
Pathological Gambling Y-BOCS (PG-YBOCS)	X	X
Subjektive Bewertung des Trainings		X

Studien haben gezeigt, dass die Vorgabe psychopathologischer Fragebögen unabhängig davon, ob diese über das Internet oder als Papier und Bleistift Version vorgegeben wurden, vergleichbare Ergebnisse liefert. Dies konnte beispielsweise für den in der vorliegenden Studie vorgegebenen BDI (Schulenberg & Yutrzenka, 2001) bestätigt werden.

4.3 Experimentelle Aufgaben

1)Deprexis

„Deprexis“ basiert auf den etablierten Techniken der Kognitiven Verhaltenstherapie (KVT) und wurde um weitere Therapiebausteine aus der Interpersonellen Therapie, aus problemlösenden sowie achtsamkeits- und akzeptanzorientierten Verfahren erweitert. Die Intervention besteht aus 11 Modulen, in denen ein therapeutisches Gespräch zwischen einem Patienten und einem Experten simuliert wird, wobei der Verlaufspfad des Programms basierend auf den Antworten des Patienten fortwährend automatisch individuell angepasst wird.

Den Betroffenen werden unter anderem Informationen zu Depressionen und deren Behandlungsmöglichkeiten an die Hand gegeben sowie Problemlösestrategien als auch Entspannungs- und Aufmerksamkeitsübungen vermittelt. Zudem lernt der Nutzer, negativ getönte Denkmuster zu modifizieren, seinen Aktionsradius wieder zu vergrößern und Rückfällen vorzubeugen. In ersten randomisierten Studien, an denen auch unsere Arbeitsgruppe beteiligt war (Meyer et al., 2009; Moritz et al., 2012), konnte die Wirksamkeit des Programms bestätigt werden: in der Interventionsgruppe konnte im Vergleich zur Wartekontrollgruppe eine signifikant (überzufällig) stärkere Reduktion der depressiven Symptomatik erzielt werden. Die Verbesserung der depressiven Symptome wurde über einen Zeitraum von sechs Monaten aufrechterhalten. „Deprexis“ hatte auch positive Auswirkungen auf das soziale Funktionsniveau (Meyer et al., 2009): So beurteilten 80% der Nutzer das Programm als hilfreich, wobei ungefähr ein Viertel der Teilnehmer klinisch signifikante Verbesserungen der Depression erfuhren und nach dem Programm keine depressiven Symptome mehr aufwiesen. Das Programm wurde im Rahmen vorheriger Studien (z.B. Moritz et al., 2012) von der Ethikkommission der Deutschen Gesellschaft für Psychologie (DGPs) als unbedenklich bewertet. Bei Menschen, die zu problematischem Glücksspiel neigen, liegen bisher noch keine wissenschaftlichen Erkenntnisse vor, die einen Rückgang des problematischen Verhaltens und der depressiven Symptome belegen. Da häufig depressive Störungen dem pathologischen Spielverhalten vorausgehen (z.B. Blaszczynski & Nower, 2002; Lorains et al., 2011), soll die Wirksamkeit von deprexis bei pathologischem Glücksspiel im Rahmen der vorliegenden Studie untersucht werden.

2) Retraining

Um darüber hinaus dem Umstand Rechnung zu tragen, dass eine weitere Subgruppe von Betroffenen primär aus Gründen des „excitement“ („Action-Spieler“) spielt, soll mit dem Retraining eine weitere Intervention zum Einsatz kommen, die im letzten Jahr für viel Aufsehen bei Wissenschaftlern gesorgt hat und die primär eine Reduktion des Spielverhaltens anstrebt. Die geplante Intervention basiert auf dem sog. Approach-Avoidance Task (Rinck & Becker, 2007), der neben der erfolgreichen Nutzung in der Diagnostik in ersten Studien auch als Trainingsprogramm eingesetzt werden konnte.

Basierend auf der wechselseitigen Verbindung von Wertigkeit und Verhalten wurde der AAT bei Abhängigkeitserkrankungen für das gezielte „Umtrainieren“ (sog. Retraining) dysfunktionaler Verhaltenstendenzen eingesetzt (für Befunde zu Alkoholabhängigkeit: Wiers, Eberl, Rinck, Becker, & Lindenmeyer, 2011; Wiers, Rinck, Kordts, Houben, K., & Strack, 2010; für Befunde zu Tabakabhängigkeit: Wittekind et al., eingereicht). Beim Einsatz als Trainingsprogramm zielt das Verfahren darauf ab, durch das gezielte Wegdrücken der störungsspezifischen Reize eine Abschwächung der assoziativen Verbindungen zwischen dem Suchtmittel und positiver Valenz zu bewirken, die wiederum in einer Abnahme des Problemverhaltens resultieren sollte. Bei der Retrainings-Variante werden dem Teilnehmer Bilder unterschiedlicher Valenz (Glücksspiel-relevante vs. neutrale Bilder) in randomisierter Reihenfolge präsentiert. Die Aufgabe besteht darin, die Bilder in Abhängigkeit einer wertneutralen Dimension (farbige Umrandung der Bilder) über das heranziehen oder wegschieben der Computermaus entweder zu sich heranzuziehen (Armflexion) oder von sich wegzuschieben (Extension). Die Bewegungsrichtung der Bilder ist an eine Zoom-Funktion gekoppelt, beim Heranziehen werden die Bilder größer, beim Wegschieben kleiner. Auf diese Weise wird die Annäherungs- bzw. Vermeidungsbewegung optisch unterstützt. Jeder Durchgang ist dabei wie folgt aufgebaut: zunächst erscheint in der Bildschirmmitte für 500ms ein Fixationskreuz, gefolgt von einem störungsspezifischen oder einem neutralen Bild. In Abhängigkeit von der farblichen Umrandung des Bildes muss dieses mit der Maus herangezogen oder weggeschoben werden. Bei der Anwendung bei pathologischem Glücksspiel wird die Instruktion so gewählt, dass störungsrelevante Stimuli weggeschoben und neutrale Bilder herangezogen werden müssen. Wird das Bild in die richtige Richtung bewegt, verschwindet es vom Bildschirm. Um mit dem nächsten Durchgang zu beginnen, ist es erforderlich, einen zentral auf dem Bildschirm ausgerichteten Knopf zu drücken, so dass sichergestellt wird, dass die Maus wieder in ihre Ausgangsposition bewegt wird. Die Bedingung sieht keine Verstärkung des schädlichen Verhaltens vor.

Bei Alkoholabhängigkeit/starkem Trinkverhalten konnte gezeigt werden, dass das Wegdrücken alkoholrelevanter Reize in einer Zunahme der Vermeidungstendenzen gegenüber diesen Reizen resultierte (z.B. Wiers et al., 2011; Wiers et al., 2010). Darüber hinaus konnte in einigen Studien sogar ein Effekt auf das Verhalten nachgewiesen werden: Teilnehmer, die Alkohol-/Tabak-relevante Reize vermeiden mussten (d.h. im AAT von sich wegdrücken mussten), zeigten eine Reduktion des Suchtmittelkonsums (Wiers et al., 2011; Wittekind et al., eingereicht). Beispielsweise konnten wir im Rahmen einer Online-Intervention, in der wir eine abgewandelte Form des AAT eingesetzt haben, in der Interventionsgruppe eine signifikante Reduktion des Zigarettenkonsums von 15% gegenüber der Wartekontrollgruppe erzielen (Wittekind et al., eingereicht).

3) aktive Kontrollintervention

Bei der aktiven Kontrollbehandlung erhalten die Teilnehmer die „klassische“ Version des AAT, bei der 50% der störungsspezifischen Bilder herangezogen und 50% der störungsspezifischen Bilder weggeschoben werden müssen.

4) Wartekontrollgruppe
Es handelt sich um eine Warte-Kontrollgruppe. Die Kontrollgruppe erhält beide Trainingsprogramme nach Ende der Abschlussuntersuchung (d.h. nach acht Wochen).

4.4 Durchführung
Sowohl die Rekrutierung als auch die Datenerhebung erfolgen anonym über das Internet. Dabei sollen nur Internetforen genutzt werden, die spezifisch für pathologisches Glücksspiel sind. Werden die Teilnehmer Deprexis zugewiesen, erhalten Sie einen Zugang zum Programm, während die Teilnehmer des Retraining herunterladen. Beide Programme werden vom bzw. auf dem heimischen Computer durchgeführt.

4.5 Körperliche Beanspruchung
Körperliche Überbeanspruchung kann ausgeschlossen werden.

4.6 Mentale Beanspruchung
Wir gehen zu jedem Befragungszeitpunkt von einer maximalen Bearbeitungszeit von 45 Minuten aus. Bei der Durchführung beider Interventionsprogramme können die Teilnehmer nach eigenem Ermessen Pausen einlegen, so dass eine Überanstrengung oder Ermüdung ausgeschlossen werden kann. Unzumutbare aversive Reize werden nicht vorgegeben.

4.7 Preisgabe persönlicher Informationen
Da es sich um eine Effektivitätsuntersuchung handelt, in der die Wirksamkeit zweier Intervention evaluiert werden soll, ist es erforderlich, soziodemographische und störungsspezifische Daten sowie das Vorliegen einer psychiatrischen/psychologischen Diagnose zu erheben. Es werden über die absolut erforderlichen Angaben hinaus keine persönlichen Daten erfragt. Die Teilnehmer müssen weder Namen noch Adressdaten angeben (siehe auch 5.1).

4.8 Täuschung und Aufklärung
Entfällt.

5. Angaben zu Aufzeichnung, Aufbereitung, Speicherung und Löschung der Daten

5.1 Personenbezogene Daten
Alle Teilnehmer werden darauf hingewiesen, dass die bei dieser Studie erfassten Daten in anonymisierter Form verarbeitet und veröffentlicht werden. Die Anonymität der Betroffenen ist gewährleistet, zumal die nicht anonym agierenden Versuchsleiter über Email im Falle von Fragen erreichbar sind (für Hinweise auf eine Anonymisierung der Email-Adresse siehe unten). So ist eine Begleitung der Teilnehmer möglich, ohne dass diese sich zu erkennen geben müssen. Die Eingaben werden beim Ausfüllen automatisch registriert und können direkt als SPSS-Datei heruntergeladen werden. *IP Adresssen werden nicht gespeichert*. Namen und Adressdaten (inkl. Bundesland) werden nicht erfragt. Um die Gruppen bzgl. wichtiger soziodemografischer Daten vergleichen zu können, werden das Geschlecht und das Alter der Betroffenen erfragt. Um differenziert untersuchen zu können, ob die Wirksamkeit eines der beiden Programme von der Medikation der Teilnehmer beeinflusst wird, wird auch die aktuelle Medikamenteneinnahme erfragt. Weitere personenbezogenen Daten werden nicht erhoben. Am Ende der Online-Erstbefragung hinterlassen die Teilnehmer ein Code-Wort, damit die in der Post-Untersuchung erhobenen Daten den prä-Daten zugeordnet werden können. Die Erstellung des Codewortes erfolgt anhand der von Ihnen vorgeschlagenen Kriterien. Die Teilnehmer werden auf

Möglichkeiten hingewiesen, kostenlose Emailadressen einzurichten, die keinen Hinweis auf die Identität liefern (z.B. sonnenblume83@gmx.de statt Hans.Maier83@gmx.de).

5.2 Datenschutz

Die Auswertung und Nutzung der Daten durch den Studienleiter und seine Mitarbeiter erfolgt in anonymisierter Form. Eine Weitergabe der erhobenen Daten im Rahmen der Studie erfolgt nur in anonymisierter Form. Gleiches gilt für die Veröffentlichung der Daten. Alle gesammelten Daten werden auf Passwort geschützten Rechnern gespeichert, das nur dem jeweiligen Computernutzer bekannt ist. Persönliche Daten werden auf Rechnern gespeichert, die keinen Internetanschluss besitzen. CD-ROMs für Festplattenspeicherungen werden in einem Tresor aufbewahrt, dessen Code nur den festen Mitarbeitern der Abteilung bekannt ist. Unsere Klinik ist zertifiziert und die Sicherheitsstandards werden über den Germanischen Lloyd sowie interimsmäßige interne Audits regelmäßig überwacht. Alle Projektmitarbeiter sind diesbezüglich geschult. Sofern Probanden die Bereitschaft äußern, an weiteren Studien teilzunehmen, holen wir hierfür eine schriftliche, jederzeit widerrufbare Einverständniserklärung ein und nutzen diese Daten nur intern. Es erfolgt keine Weitergabe an Dritte.

5.3 Kodierliste und persönliches Codewort

Wie bereits unter 5.2 dargestellt, werden die Daten auf passwortgeschützten Rechnern gespeichert. Die Zuordnung der Daten aus der prä- und post Befragung erfolgt über ein Codewort, das anhand der von der Ethikkommission der DGPs vorgeschlagenen Kriterien erstellt wird.

5.4 Löschung der Daten

Die anonymisierten Daten werden nach Abschluss der Studie für zehn Jahre archiviert. Im Falle des Widerrufs der Einverständniserklärung werden die bereits erhobenen Daten gelöscht. Nach Abschluss der Studie werden alle Emailadressen gelöscht. Danach ist ein Widerruf der Daten nur noch bei Nennung des persönlichen Codewortes möglich.

6. Gewinnung der Personenstichprobe und Vergütung von Probanden

6.1 Rekrutierung

Um die Wirksamkeit der Interventionen zu evaluieren, sollen Personen mit pathologischem Glücksspielverhalten über qualitativ hochwertige Online-Foren (Kriterien: Spezialisierung des Forums auf pathologisches Glücksspiel, Forum wird von einem Moderator betreut, Teilnahme am Forum nur nach Anmeldung) rekrutiert werden. Vor Studienaufruf in Online-Foren wird zunächst der Webmaster des entsprechenden Forums kontaktiert, um den Studienaufruf genehmigen zu lassen.

<u>Fallzahlkalkulation</u>: Die Berechnung der optimalen Stichprobengröße wurde mit der Statistiksoftware gpower® durchgeführt. Die Powerberechnungen haben ergeben, dass bei einer mittleren Effektstärke von f = .25 (mittlerer Effekt), dass bei einem α = .05 und einem ß = .80 eine 45 Teilnehmer je Gruppe rekrutiert werden müssen (Gesamtstichprobe: 180). Erfahrungen zufolge ist mit einem Dropout von etwa 20% zu rechnen, die auf die Stichprobengröße aufgeschlagen werden. So ergibt sich eine jeweilige Gruppengröße von 54 Teilnehmern (Gesamtstichprobe: 216).

6.1.1 Statistische Analysen

Für die Auswertung wählen wir eine intention-to-treat Analyse, in der alle Teilnehmer berücksichtigt werden, die an der online-Erstbefragung teilgenommen haben. Fehlende Werte zu t1 werden über zwei Methoden überprüft: 1) *last observation carried forward* (LOCF; führt zu

tendenziell konservativen Entscheidungen), 2) Multiple imputation (führt zu tendenziell progressiven Entscheidungen). Weiterhin ist eine per protocol (PP) Analyse vorgesehen, die nur die Completer einschließt. Ebenfalls auf Anraten Prof. Wegscheiders vergleicht die primäre statistische Analyse die Post-Ergebnisse zwischen den Gruppen mit Hilfe einer Kovarianzanalyse (ANCOVA). Mit dem Baseline-Wert (Ergebnisse zum prä-Zeitpunkt) als Kovariate wird u.a. eine mögliche Regression zur Mitte bereinigt, was über konventionelle zweifaktorielle Analysen nicht erreicht werden kann. In einer Subgruppenanalyse sollen zudem vorbehandelte Patienten mit bislang unbehandelten Betroffenen hinsichtlich der Outcomeparamter verglichen werden. Auch mögliche Prädiktorvariablen, wie beispielsweise die Schwere der Symptomatik, werden bei der Auswertung berücksichtigt.

6.2 Personenstichprobe aus Datenbank
Es ist nicht vorgesehen, die Teilnehmer über eine bestehende Probandendatenbank zu rekrutieren.

6.3 Merkmale der Personenstichprobe
Es werden nur Teilnehmer zwischen 18 und 65 Jahren eingeschlossen. Für die Beschreibung der Population siehe 6.1.

6.4 Einschluss- und Ausschlusskriterien
Einschlusskriterien:

- Alter: 18 bis 65 Jahre
- Einwilligungsfähigkeit (informed consent)
- Vorliegen eines Internetzugangs
- Ausreichendes Verständnis der deutschen Sprache
- Bereitschaft, an zwei anonymen Online-Befragungen teilzunehmen, welche jeweils ca. 25-30min dauern
- Bereitschaft, an einem 8-wöchigen Trainingsprogramm teilzunehmen
- Bereitschaft, die Programme auf eigene Verantwortung zu nutzen.
- Bereitschaft, eine Email-Adresse zu hinterlassen
- Vorliegen einer mindestens moderat ausgeprägten depressiven Symptomatik (> 11 Punkte im BDI-II)

Ausschlusskriterien:

- Vorhandensein einer lebenszeitlichen schizophrenen oder bipolaren Symptomatik
- Das Vorhandensein von akuten suizidalen Tendenzen

6.5 Internetbasierte Datengewinnung
Die Erhebung der Daten findet ausschließlich über das Internet statt. Für das Vorgehen bei online-Studien haben wir uns eingehend vom Datenschutzbeauftragten der Stadt Hamburg (Dr. Menzel) beraten lassen; die Empfehlungen und Vorgaben für frühere Studien wurden auch in diesem Antrag berücksichtigt (z.B. werden IP-Adressen nicht gespeichert). Wie unter 5. ausführlich dargelegt, werden die Teilnehmer explizit auf die Möglichkeit der Anonymisierung informiert. Die besonderen datenschutzrechtlichen Anforderungen sind dem Antragsteller aus vorherigen Erfahrungen bekannt und wurden bereits mehrfach erfolgreich bewältigt. Zu Beginn der Onlinebefragung werden die Ein- und Ausschlusskriterien abgefragt. Erfüllt ein Teilnehmer die Ausschlusskriterien, wird in Unipark eine „Falltür“ eingebaut, so dass der Teilnehmer automatisch von der weiteren Befragung ausgeschlossen wird. Der Teilnehmer wird auf eine neue Seite geleitet, in dem die Gründe des Ausschlusses kurz und nicht-stigmatisierend dargelegt werden. Bei Fragen können die Teilnehmer jederzeit den Studienleiter kontaktierten. Das

Vorgehen ist mit der Datenschutzbehörde der Stadt Hamburg abgestimmt. Herr Dr. Menzel hat unser Procedere ausdrücklich befürwortet. Wir möchten nochmals betonen, dass die Daten auf einem sicheren Server gespeichert werden und die IP-Adresse nicht gespeichert wird.
Des Weiteren wurden über das Internet durchgeführte Therapieevaluationen bereits in international anerkannten Fachzeitschriften veröffentlicht und von den entsprechenden Ethikkommissionen ebenfalls als unbedenklich eingestuft (z.B. Depression: Andersson et al., 2005; Panikstörung: Carlbring, Ekselius, & Andersson, 2003). Auch unsere Arbeitsgruppe verfügt über langjährige Erfahrungen mit Internetstudien, die z.T. hochrangig publiziert wurden (Jelinek, Hottenrott, & Moritz, 2009; Moritz & Jelinek, 2009; Moritz & Laroi, 2008) und von der jeweiligen Ethikkommission als unbedenklich erachtet wurden.

6.6 Teilnahmevergütung
Die Teilnehmer erhalten außer der kostenlosen Teilnahme keine finanzielle Aufwandsentschädigung.

7. Freiwilligkeit der Teilnahme und Rücktritt
Durch die Durchführung der Studie als Online-Intervention ist sichergestellt, dass die Teilnahme auf Freiwilligkeit basiert. Die Teilnehmer werden im Vorfeld der Teilnahme ausführlich über Sinn und Zweck der geplanten Untersuchung informiert. Sie müssen der Teilnahme explizit zustimmen, bei Ablehnung wird die Online-Erstbefragung nicht gestartet und es werden keinerlei Daten erhoben.
Auch im weiteren Verlauf der Studiendurchführung ist entsprechend der Deklaration von Helsinki (vgl. dazu Ziff. I 5, 6, II. 4 und 6 der revidierten Deklaration von Helsinki; § 40 (1) 1. AMG, § 20 (1) 1. MPG) gewährleistet, dass zu jedem Zeitpunkt der Studie die Belange der Probanden Vorrang haben. Bei Nicht-Einwilligung erwachsen den Patienten keinerlei Nachteile. Ebenso kann die Einwilligung zu dieser Studie zu jedem Zeitpunkt zurückgezogen werden. Etwaige Risiken bei einem Abbruch sind zu keinem Zeitpunkt zu erwarten. Im Rahmen der Teilnehmeraufklärung und -einwilligung vor Beginn der Studie wird die Vereinbarung getroffen, dass die Teilnehmer der Warte-Kontrollgruppe nach Studienende das *Retrainings*-Programm erhalten.

8. Umgang mit auffälligen Befunden
8.1 Aufklärung
Entfällt.

8.2 Teilnahmebeschränkung
Entfällt.

9. Informiertheit und Einwilligung
9.1 Informiertheit
Die Teilnehmer werden vollständig über die Studienzwecke, die Intervention, die Ein- und Ausschlusskriterien sowie Untersuchungsverlauf aufgeklärt (s. Teilnehmerinformation).

9.2 Einwilligung
Die Probanden werden darauf hingewiesen, dass ihre Teilnahme an der Studie freiwillig erfolgt, und dass sie ihr Einverständnis jederzeit ohne Angabe von Gründen und ohne weitere Folgen zurückziehen können (siehe Teilnehmerinformation). Eine Teilnahme an dieser Studie ist erst nach schriftlicher Einwilligung durch Zustimmung zur Einwilligungserklärung (s. Anhang) möglich. Im Gegensatz zu nicht-internetbasierten Studien, in denen die persönliche schriftliche Einwilligung der Studienteilnehmer üblicherweise gefordert wird, ist der Einsatz eines

elektronischen bzw. Online-basierten „Informed Consent“ und Einwilligungs-Prozedere geplant. Das Verfahren des Informed Consent/Online-Einwilligung wurde in vorherigen Studien ausführlich mit der Datenschutzbehörde besprochen und von zuständigen Ethikkommissionen als ethisch unbedenklich bewertet und soll im Rahmen dieser Studie abermals eingesetzt werden.

9.3 Bild- und Tonaufnahmen
Entfällt.

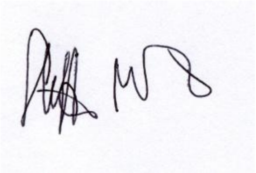

Prof. Dr. Steffen Moritz (Antragsteller)
(elektronische Unterschrift)

Literatur

Andersson, G., Bergstrom, J. A. N., Hollandare, F., Carlbring, P. E. R., Kaldo, V., & Ekselius, L. (2005). Internet-based self-help for depression: randomised controlled trial. *The British Journal of Psychiatry, 187*, 456-461.

Blaszczynski A., & Nower L. (2002). A pathways model of problem and pathological gambling. *Addiction* 97, 487–99.

Carlbring, P., Ekselius, L., & Andersson, G. (2003). Treatment of panic disorder via the Internet: A randomized trial of CBT vs. applied relaxation. *Journal of Behavior Therapy and Experimental Psychiatry, 34,* 129-140.

Hand, I. (2004). Negative und positive Verstärkung bei pathologischem Glücksspielen: Ihre mögliche Bedeutung für die Theorie und Therapie von Zwangsspektrumsstörungen. *Verhaltenstherapie, 14*, 133-144.

Jelinek, L., Hottenrott, B., & Moritz, S. (2009). When cancer is associated with illness but no longer with animal or zodiac sign: investigation of biased semantic networks in obsessive-compulsive disorder (OCD). *Journal of Anxiety Disorders, 23*, 1031-1036.

Lorains, F. K., Cowlishaw, S., & Thomas, S. A. (2011). Prevalence of comorbid disorders in problem and pathological gambling: systematic review and meta-analysis of population surveys. *Addiction,* 106, 490-498.

Meyer, B., Berger, T., Caspar, F., Beevers, C. G., Andersson, G., & Weiss, M. (2009). Effectiveness of a novel integrative online treatment for depression (Deprexis): Randomized controlled trial. *Journal of Medical Internet Research*, 11, e15.

Moritz, S., & Jelinek, L. (2009). Inversion of the "unrealistic optimism" bias contributes to overestimation of threat in obsessive-compulsive disorder. *Behavioural and cognitive psychotherapy, 37*, 179-193.

Moritz, S., & Larøi, F. (2008). Differences and similarities in the sensory and cognitive signatures of voice-hearing, intrusions and thoughts. *Schizophrenia Research, 102*, 96-107.

Moritz, S., Schilling, L., Hauschildt, M., Schröder, J., & Treszl, A. (2012). A randomized controlled trial of internet-based therapy in depression. *Behaviour Research and Therap*y, 50, 513-521.

Rinck, M., & Becker, E. S. (2007). Approach and avoidance in fear of spiders. *Journal of Behavior Therapy and Experimental Psychiatry, 38*, 105-120.

Schulenberg, S. E., & Yutrzenka, B. A. (2001). Equivalence of computerized and conventional versions of the Beck Depression Inventory-II (BDI-II). *Current Psychology, 20*, 216-230.

Wiers, R. W., Eberl, C., Rinck, M., Becker, E. S., & Lindenmeyer, J. (2011). Retraining automatic action tendencies changes alcoholic patients' approach bias for alcohol and improves treatment outcome. *Psychological Science, 22*, 490-497.

Wiers, R. W., Rinck, M., Kordts, R., Houben, K., & Strack, F. (2010). Retraining automatic action-tendencies to approach alcohol in hazardous drinkers. *Addiction, 105*, 279-287.

Universitätsklinikum Hamburg-Eppendorf

Zentrum für Psychosoziale Medizin
Prof. Dr. Dieter Naber
Ärztlicher Leiter

Klinik und Poliklinik für Psychiatrie und Psychotherapie
Arbeitsgruppe Klinische Neuropsychologie
Martinistraße 52
20246 Hamburg
Ansprechpartner für eventuelle Rückfragen:
Prof. Dr. Steffen Moritz
moritz@uke.uni-hamburg.de
Telefon: (040) 7410-56565
Telefax: (040) 7410-57566

Allgemeine Teilnehmerinformation über die Untersuchung
Universitätsklinikum Hamburg-Eppendorf, Arbeitsgruppe Klinische Neuropsychologie

Titel der Studie:
„Computerbasiertes Trainingsprogramm für Menschen mit Glücksspielproblemen“

Sehr geehrte/r Studieninteressent/in,

wir möchten Sie zu einer wissenschaftlichen Online-Studie einladen. Ziel der Untersuchung ist es, die Wirksamkeit von Selbsthilfe (im Rahmen eines computerbasierten Trainings) auf Ihr Wohlbefinden und problematisches Spielverhalten zu überprüfen. Bevor Sie sich für eine Studienteilnahme entscheiden, möchten wir Sie ausführlich über Sinn und Zweck der geplanten Studie sowie deren Ablauf informieren. Bitte lesen Sie sich die nachfolgenden Informationen sorgfältig durch, bevor Sie sich für eine Studienteilnahme entscheiden. Bei Fragen oder Unklarheiten können Sie sich gerne per Email an die Versuchsleiterin Frau Dipl.-Psych. Charlotte Wittekind (c.wittekind@uke.uni-hamburg.de) wenden.

Ablauf der Studie

Was ist das Ziel der Studie?

Wir sind Wissenschaftler des Universitätsklinikums Hamburg-Eppendorf (UKE) und beschäftigen uns seit Jahren mit der Erforschung, Diagnostik und Therapie psychischer Störungen und Verhaltensauffälligkeiten. In den letzten Jahren haben wir verstärkt zu Selbsthilfeansätzen bei verschiedenen Störungsbildern geforscht. Aktuell möchten wir die Wirksamkeit computerbasierten Trainings bei pathologischem Glücksspiel untersuchen. Für viele Personen ist Glücksspiel nur ein harmloser Freizeitvertreib, dem Sie z.B. einmal im Jahr mit kleinen Geldbeträgen nachgehen. Für andere entstehen dadurch große finanzielle Probleme und Konflikte mit Familie und Partnern. Obwohl wirksame Therapieangebote bestehen, nimmt nur eine Minderheit der Betroffenen (therapeutische) Hilfe in Anspruch. Als häufigste Hürden, die einer Therapie im Weg stehen, werden der Wunsch, das Problem eigenständig zu lösen, Scham/Verlegenheit oder Angst vor Stigmatisierung genannt. Aus diesem Grund bietet (computerbasierte) Selbsthilfe eine anonyme, alternative und leicht zugängliche Behandlungsmöglichkeit. Die Teilnahme an der Studie ist selbstverständlich kostenlos.

Wie läuft die Studie ab?

1. Nachdem Sie Ihre Einwilligungserklärung abgegeben haben, möchten wir Sie zunächst bitten, an einer Eingangsbefragung teilzunehmen, die etwa 25-30 Minuten dauert. Bei der Befragung selbst handelt es sich um eine schriftliche, anonyme Interneterhebung über die Software Unipark®. Die Daten werden online gespeichert und können direkt mit einem Statistikprogramm heruntergeladen werden. Der Zugriff auf die Daten ist Passwort-geschützt. Nur Mitarbeiter der Arbeitsgruppe können auf die Daten zugreifen, die im Übrigen keinen Rückschluss auf Ihren Rechnerstandort oder Aufenthaltsort zulassen. Die Befragung beinhaltet allgemeine Fragen zu Ihrer Person (z.B. Alter, Schulbildung), zu Ihrer derzeitigen psychischen Verfassung (z.B. Stimmung) sowie zu Ihrem Spielverhalten. Am Ende der Befragung werden Sie gebeten, eine Email Adresse und ein anonymes persönliches Code-Wort zu hinterlassen. Um absolute Anonymität zu gewährleisten, möchten wir Sie auf die Möglichkeit hinweisen, eine kostenlose Emailadresse einzurichten, die keinen Hinweis auf Ihre Identität erlaubt (z.B. sonnenblume83@gmx.de statt hans.maier83@gmx.de).
Nach dem Zufallsprinzip erhält ein Teil der Teilnehmer im Anschluss an die Online-Eingangsbefragung das Trainingsprogramm. Das Programm soll über einen Zeitraum von 8 Wochen durchgeführt werden. Der andere Teil erhält entweder ein aktives Kontrolltraining oder muss sich 8 Wochen gedulden. Nach Abschluss der Online-Nachbefragung (siehe Punkt 2) erhalten alle Teilnehmer Zugang zum Trainingsprogramm. Dieses Vorgehen ist wissenschaftlich anerkannt (sog. randomisiert-kontrollierte Studie). **Das Vorhaben wurde von der Ethikkommission der Deutschen Gesellschaft für Psychologie (DGPs) genehmigt.**

2. Nach Ablauf der 8 Wochen erhalten Sie von uns eine Nachricht, in der Sie zu einer Online-Nachbefragung aufgefordert werden. Die Nachbefragung ist von großer Bedeutung, da wir ohne Ihre erneute Teilnahme keine Aussagen über die Umsetzbarkeit und Wirksamkeit des Trainingsprogramms machen können.

Wer kann an der Studie teilnehmen?

Sie können an der Studie teilnehmen, wenn Sie die folgenden Einschlusskriterien erfüllen:

1. Sie leiden unter Glücksspielproblemen und sind bereit, an einem 8-wöchigen Trainingsprogramm teilzunehmen.
2. Sie sind zwischen 18 und 65 Jahre alt.
3. Sie erklären sich bereit, das Programm auf eigene Verantwortung zu nutzen. Zwar gibt es bisher keine Anhaltspunkte, dass das Programm schädlich sein könnte, dennoch können negative Effekte nicht vollkommen ausgeschlossen werden. Es besteht die Möglichkeit, dass Sie nicht von dem Training profitieren.
4. Sie besitzen einen Internetzugang.
5. Sie verstehen die deutsche Sprache ausreichend.
6. Sie sind bereit, an zwei anonymen Online-Befragungen teilzunehmen, welche jeweils ca. 25-30min dauern.
7. Sie sind bereit, eine Studien-Email-Adresse zu hinterlassen.
8. Sie können auch an der Studie teilnehmen, wenn Sie derzeit an anderen Therapiemaßnahmen teilnehmen. Bestehende Angebote, die Sie parallel bekommen (z.B. Psychotherapie, Pharmakotherapie) bleiben durch die Teilnahme an der Studie unangetastet.

Sie können leider nicht an der Studie teilnehmen, wenn:

1. Sie an einer bipolaren Störung oder an einer Psychose leiden.

2. Bei Ihnen akute Selbstmordgedanken und/oder –tendenzen vorliegen.
   Sollte dies der Fall sein, begeben Sie sich umgehend in ärztliche Behandlung und zögern Sie auch nicht ggf. einen Notarzt (Telefon 112) zu rufen!

Was habe ich davon, an der Studie teilzunehmen?
Zum einen bietet Ihnen die Teilnahme an der Studie die Möglichkeit, kostenlos und anonym ein Trainingsprogramm zu erhalten. Zum anderen tragen Sie durch Ihre Teilnahme dazu bei, dass Hilfsangebote für Menschen, die Probleme mit ihrem Glücksspielverhalten haben, verbessert werden können.

Freiwilligkeit und Anonymität
Die Teilnahme an der Studie ist freiwillig. Sie können jederzeit formlos und ohne Angabe von Gründen Ihre Einwilligung zur Teilnahme an dieser Studie widerrufen, ohne dass Ihnen daraus Nachteile entstehen. In diesem Fall werden alle bereits gespeicherten Daten gelöscht. Bitte haben Sie Verständnis, dass eine Löschung Ihrer Eingaben dann nicht mehr möglich ist, wenn diese bereits ausgewertet wurden, da die Daten anonym sind und nicht mehr einzelnen Personen zugeordnet werden können. Die im Rahmen dieser Studie erhobenen Daten und persönlichen Mitteilungen werden vertraulich behandelt. Alle persönlichen Angaben, die Sie uns gegenüber machen, unterliegen der ärztlichen Schweigepflicht. Auch diejenigen Mitarbeiter, die durch direkten Kontakt mit Ihnen über personenbezogene Daten verfügen, unterliegen der Schweigepflicht. Des Weiteren wird die Veröffentlichung der Ergebnisse der Studie in anonymisierter Form erfolgen, d. h. ohne dass Ihre Daten Ihrer Person zugeordnet werden können.

Datenschutz
Die Erhebung der Daten erfolgt vollständig anonymisiert, d.h. an keiner Stelle wird Ihr Name erfragt. In der Studie werden nur allgemeine Daten zur Beschreibung Ihrer Person (Alter, Geschlecht, Schulbildung), zu Ihrer psychischen Verfassung sowie zu Ihrem Spielverhalten erfragt. Wir benötigen jedoch von Ihnen eine Emailadresse, um Ihnen den Link für die Online-Nachbefragung zusenden zu können. Falls Sie eine personenbezogene Emailadresse haben, d.h. eine Emailadresse aus der z.B. Ihr Name hervorgeht, wären Rückschlüsse auf Ihre Identität möglich. Damit dies nicht möglich ist, verwenden Sie bitte eine Studien-Emailadresse mit Pseudonym (d.h. aus der Ihr Name nicht hervorgeht) oder legen Sie sich ggf. eine neue nicht-personenbezogene Emailadresse zu (über diverse Anbieter kostenlos möglich, z.B. gmx). Alle Emailadressen werden vertraulich behandelt und nicht an Dritte außerhalb der Arbeitsgruppe weitergegeben. Nach Abschluss der Studie werden alle angegebenen Emailadressen gelöscht.
Um Ihre Daten der Eingangsbefragung den Daten der Abschlussbefragung richtig zuordnen zu können, ohne die Geheimhaltung zu verletzen, benötigen wir ein persönliches Codewort, das Sie selbst anhand einer Regel erstellt haben und das außer Ihnen niemand kennt. Das persönliche Codewort erlaubt keine Rückschlüsse auf Ihre Person. Die anonymisierten Daten werden mindestens 10 Jahre gespeichert. Sie können allerdings, wenn immer Sie dies möchten, die Löschung der von Ihnen erhobenen Daten verlangen (vor der Datenauswertung). Dazu müssen Sie uns nicht Ihren Namen verraten, sondern nur Ihr Codewort. Für die Erstellung Ihres Codeworts erhalten Sie eine Anleitung „Wie erstellen Sie Ihr persönliches Codewort?“ Dieses Blatt können Sie herunterladen, so dass Ihr Codewort nicht in Vergessenheit gerät.
Die erhobenen Daten werden zusammen mit Ihrer Emailadresse und ihrem persönlichen Code-Wort ausschließlich zum Zweck der Studiendurchführung in digitaler Form gespeichert. Die Daten werden zunächst vorläufig auf einem gesicherten Server und später auf einem passwortgeschützten Rechner gespeichert. Es werden nur Daten erfragt, die für die Auswertung der Studie erforderlich sind. Dabei ist auch die Sicherheit der Datenübermittlung über das

Internet durch eine SSL-Verschlüsselung gewährleistet. Die Auswertungen können in Fachzeitschriften veröffentlicht werden, ohne Offenlegung der persönlichen Angaben. Die Datenschutzbestimmungen werden für die Verarbeitung der personenbezogenen Daten eingehalten.

Hat die Teilnahme Nachteile für mich?
Es liegen keine Hinweise dafür vor, dass das Trainingsprogramm schädlich sein könnte, dennoch können negative Effekte, wie z.B. die vorübergehende Verstärkung des Spieldranges durch die konkrete Auseinandersetzung mit der Problematik, nicht vollkommen ausgeschlossen werden. Dies ist selbstverständlich nicht beabsichtigt. Die Teilnahme an der Studie erfolgt auf eigene Verantwortung. Sollte es zu einer Verschlechterung des Spielverhaltens kommen, können Sie unter folgender Telefonnummer Unterstützung bekommen:
Beratungstelefon der Bundeszentrale für gesundheitliche Aufklärung (BZgA)
Tel: 0800 1 37 27 00

Vergütung
Für die Teilnahme an der Untersuchung erhalten Sie außer einer kostenlosen Studienteilnahme keine Aufwandsentschädigung. Am Ende erhalten alle Teilnehmer Zugang zu zwei unterschiedlichen Trainingsprogrammen (auch die Teilnehmer, die zunächst keinen Zugang erhielten).

Bei Rückfragen stehen wir Ihnen unter der Emailadresse moritz@uke.uni-hamburg.de zur Verfügung.

Mit freundlichen Grüßen,

Prof. Dr. Steffen Moritz & Dipl.-Psych. Charlotte Wittekind und
Universitätsklinikum Hamburg-Eppendorf
Klinik für Psychiatrie und Psychotherapie
Arbeitsgruppe Klinische Neuropsychologie
Martinistraße 52
D-20246 Hamburg

Prof. Dr. Iver Hand
Verhaltenstherapie Falkenried
Leiter des „Spieler-Projekts“
Falkenried 7
D-20248 Hamburg

Universitätsklinikum Hamburg-Eppendorf

Zentrum für Psychosoziale Medizin
Prof. Dr. Dieter Naber
Ärztlicher Leiter

Klinik und Poliklinik für Psychiatrie und Psychotherapie
Arbeitsgruppe Klinische Neuropsychologie
Martinistraße 52
20246 Hamburg
Ansprechpartner für eventuelle Rückfragen:
Prof. Dr. Steffen Moritz
moritz@uke.uni-hamburg.de
Telefon: (040) 7410-56565
Telefax: (040) 7410-57566

Einwilligungserklärung
Universitätsklinikum Hamburg-Eppendorf, Arbeitsgruppe Klinische Neuropsychologie

Titel der Studie:
„Computerbasiertes Trainingsprogramm für Menschen mit Glücksspielproblemen“

Ich bin bereit, an der Wirksamkeitsstudie des computerbasierten Trainingsprogramms teilzunehmen.

1. Ich bestätige, dass ich Kenntnis über die schriftliche Studieninformationen genommen habe. Ich bin über die Ziele, den Ablauf sowie über die möglichen Vor- und Nachteile einer Teilnahme an der Studie informiert und habe alles verstanden. Weiterhin ist mir bewusst, dass neben dem Programm noch andere Behandlungsmöglichkeiten von pathologischem Spielverhalten bestehen. Mir ist bewusst, dass während der Studienteilnahme auch andere Therapien in Anspruch genommen werden können.
2. Meine Fragen im Zusammenhang mit der Teilnahme an dieser Studie sind durch die erhaltenen Informationen beantwortet worden. Ich weiß, dass ich mich für weitere Fragen jederzeit über Email an die Studienleitung – Herrn Prof. Dr. Steffen Moritz – wenden kann (moritz@uke.uni-hamburg.de).
3. Ich bin bereit, an einer schriftlichen Befragung über das Internet zu zwei Zeitpunkten teilzunehmen.
4. Mit der beschriebenen Handhabung der erhobenen Daten bin ich einverstanden. Die Aufzeichnung und Auswertung der Daten erfolgt anonymisiert, d. h. unter Verwendung eines persönlichen Codewortes, das ich selbst erstellt habe und das nur ich kenne. Die Anleitung, wie das Codewort erstellt wird, erhalte ich am Ende der Online-Eingangsbefragung.
5. Mir ist bekannt, dass ich mein Einverständnis zur Aufbewahrung bzw. Speicherung meiner Daten widerrufen kann, ohne dass mir daraus Nachteile entstehen. Ich bin darüber informiert worden, dass ich jederzeit eine Löschung all meiner Daten (bis zur Datenauswertung) verlangen kann. Ich bin einverstanden, dass meine anonymisierten Daten zu Forschungszwecken weiter verwendet werden können und mindestens 10 Jahre gespeichert bleiben.
6. Ich hatte genügend Zeit für eine Entscheidung und bin bereit, an der o.g. Studie teilzunehmen. Ich weiß, dass die Teilnahme an der Studie freiwillig ist und ich die Teilnahme jederzeit ohne Angaben von Gründen beenden kann. Ich habe zur Kenntnis genommen, dass nicht vollkommen ausgeschlossen werden, dass ich persönlich vielleicht nicht von dem Angebot profitiere oder dass - obwohl bisher nicht bekannt - es vorübergehend zu einer Verschlechterung der Befindlichkeit kommt. Mir ist bekannt, dass keine Versuchspersonenversicherung besteht.

Bei Fragen oder anderen Anliegen kann ich mich an folgende Personen wenden:

<u>Versuchsleiterin:</u><br>Dipl.-Psych. Charlotte Wittekind<br>Martinistr. 52<br>20246 Hamburg<br>040-7410-53522<br>c.wittekind@uke.de	<u>Projektleiter:</u><br>Prof. Dr. Steffen Moritz<br>Martinistr. 52<br>20246 Hamburg<br>040-7410-56565<br>moritz@uke.de

Einwilligungserklärung (entsprechend des Telemediengesetzes § 13, Absatz (2)):

- o „Ja, ich stimme der Studienteilnahme zu, alle unter 1 bis 8 genannten Punkte treffen auf mich zu."
- o „Nein, ich stimme der Studienteilnahme nicht zu; ein oder mehrere der unter 1 bis 8 genannten Punkte treffen nicht auf mich zu."

Universitätsklinikum Hamburg-Eppendorf

Zentrum für Psychosoziale Medizin
Prof. Dr. Dieter Naber
Ärztlicher Leiter

Klinik und Poliklinik für Psychiatrie und Psychotherapie
Arbeitsgruppe Klinische Neuropsychologie
Martinistraße 52
20246 Hamburg
Ansprechpartner für eventuelle Rückfragen:
Prof. Dr. Steffen Moritz
moritz@uke.uni-hamburg.de
Telefon: (040) 7410-56565
Telefax: (040) 7410-57566

Wie erstellen Sie Ihr persönliches Codewort?

Um Ihre Daten richtig zuordnen zu können, ohne die Geheimhaltung zu verletzen, benötigen wir ein Kenn- oder Codewort. Das Codewort ist so aufgebaut, dass niemand von Ihrem Codewort auf Ihre Person rückschließen kann, auch wir nicht. Sie selbst können Ihr Codewort aber jederzeit rekonstruieren, wenn Sie danach gefragt werden und es vergessen haben sollten. Wir brauchen Ihnen nur die Regel zu verraten, nach der Sie es herstellen müssen.

Aus diesen Teilen besteht Ihr Codewort:

1. der Anzahl der Buchstaben des *Vornamens* Ihrer Mutter
2. den beiden letzten Buchstaben des Mädchennamens (*Geburtsnamens*) Ihrer Mutter
3. den beiden letzten Buchstaben des *Vornamens* Ihres Vaters
4. Ihrem eigenen Geburtstag (nur dem Tag, nicht Monat und/oder Jahr)

* Bitte schreiben Sie alle Zahlen zweistellig, d.h. wenn nötig mit führender Null.
* Bei mehreren oder zusammengesetzten Vornamen berücksichtigen Sie bitte nur den ersten.
* Wenn Sie den jeweiligen Namen nicht kennen, schreiben Sie statt der Buchstaben XX bzw. für die Zahl 00.

Beispiel (fiktiv)

Name der Mutter:	**Elke-**Hannelore Müller geb. Mayerhof**er**
Name des Vaters:	Wo**lf**-Rüdiger Müller
Ihr Geburtstag:	**09**.11.1987
Daraus ergibt sich als Codewort:	**04ERLF09**

Bitte tragen Sie jetzt in die Kästchen Ihr Codewort ein:

Die beiden letzten Buchstaben des Geburtsnamens Ihrer Mutter:	… …
Die Anzahl der Buchstaben des (ersten) Vornamens Ihres Mutter:	… …
Die beiden letzten Buchstaben des (ersten) Vornamens Ihres Vaters:	… …
Ihr eigener Geburtstag (nur der *Tag*):	… …

Wichtig: Diese Anleitung verbleibt bei Ihnen. Sie sollten sie niemandem zeigen!
