# Supplementary material for: Effects of a depression-focused internet intervention in slot machine gamblers: A randomized controlled trial
Source: PLoS One. 2018 Jun 8;13(6):e0198859. doi: 10.1371/journal.pone.0198859 (PMC5993308; doi:10.1371/journal.pone.0198859)
Supplement: S1 Translation — (DOCX) [file pone.0198859.s005.docx]

**Translation of relevant parts of the request for the opinion of the Ethics Committee of the DGPs**

**1. Name of the research project**

"Computer-based training program for people with gambling problems."

**(…)**

**3. Information on the framework conditions of the project**

This is a project funded by the slot-machine industry. An opinion of the Ethics Committee is recommended.

**4. Subject matter and procedure of the project**

**4.1 Subject matter**

The study investigates the effectiveness of computer-based training programs in patients with pathological gambling. Existing or parallel therapies remain untouched by the study. The main objective of the study is to investigate the extent to which two experimental study arms (“Deprexis” and retraining) lead to a differentially greater reduction of pathological gambling than an active control intervention or a waiting list control condition. Both the Deprexis and retraining programs are self-help programs that have already been tested for their effectiveness (Deprexis for the reduction of depressive symptoms; retraining for the reduction of dependency disorders) and have been classified as harmless in previous studies by the Ethics Committee of the German Society of Psychology (DGPs). It is expected that the two intervention programs will lead to a significant reduction of the pathological gaming behavior (measured by PG Y-BOCS) compared to the active control and waiting control condition with a clear advantage for the "causal therapy" (Deprexis). Analogous to Hand (2004) it is expected that "retraining", which can be described as a symptom method, is particularly effective for "positive amplifiers" (especially addiction to entertainment/changing; action-seeker), while Deprexis is more effective for so-called "negative amplifiers" (people with primary psychological (e. g. affective) disorders; escape-seeker).

**4.2 Methods**

(…) Two dates of investigation are envisaged, with the intervention period (pre-post) being eight weeks. Sociodemographic and psychopathological data are collected in an internet survey with the software Unipark®. Table 1 shows the instruments provided for the two data collection dates. At the end of the study, all participants will receive self-help manuals from our working group to promote study coherence. Participation in the study is also possible, if other therapies (e. g. psychopharmaco- or psychotherapy) are already underway.

**Tabel 1**. Survey instruments for the two assessment times

| **Instrument** | **Baseline** | **Post**  **(8 weeks)** |
| --- | --- | --- |
| Sociodemographic questionnaire | X | X |
| Short anamnesis | X |  |
| *General psychopathology* |  |  |
| Beck-Depressions Inventar (BDI-II)  Suicide Behaviors Questionnaire-Revised (SBQ-R)  Generalized Anxiety Disorder 7-Item (GAD-7)  Alcohol Use Disorders Identification Test (AUDIT) | X  X  X  X | X  X  X  X |
| *Questionnaires on different aspects of pathological gambling* |  |  |
| Questions on different types of gambling  Questions on Gambling behavior  South Oaks Gamling Scale (SOGS)  Gambling Attitudes and Beliefs Scale (GABS)  Kurzfragebogen zum Glücksspielverhalten (KFG)  Pathological Gambling Y-BOCS (PG-YBOCS) | X  X  X  X  X  X | X  X  X  X |
| Subjektive appraisal of the training |  | X |

Studies have shown that the specification of psychopathological questionnaires, regardless of whether they were given over the Internet or as a paper-pencil version, produce comparable results. This could be confirmed, for example, for the BDI (Schulenberg & Yutrzenka, 2001) specified in the present study.

**4.3 Experimental Tasks**

1) Deprexis

Deprexis is based on established techniques of Cognitive Behavioral Therapy (CBT) and has been extended by further therapy modules from interpersonal therapy, problem-solving as well as mindfulness and acceptance-oriented procedures. The intervention consists of 11 modules in which a therapeutic conversation between a patient and an expert is simulated, whereby the pathway of the program is continuously and automatically adapted individually based on the patient's responses.

Those affected are provided with information on depression and its treatment options, problem solving strategies as well as relaxation and attention exercises. In addition, the user learns to modify negative-toned thought patterns, to increase his radius of action again and to prevent relapses. In the first randomized studies, in which our working group was also involved (Meyer et al., 2009; Moritz et al., 2012), the effectiveness of the program was confirmed: in the intervention group a significant (randomly) greater reduction of depressive symptoms could be achieved compared to the control group. The improvement in depressive symptoms was maintained over a period of six months. Deprexis also had a positive impact on the social function level (Meyer et al., 2009): 80% of users rated the program as helpful, with about a quarter of the participants experiencing clinically significant improvements in depression and no depressive symptoms after the program. The program has been assessed as harmless by the Ethics Commission of the German Society of Psychology (DGPs) in previous studies (e. g. Moritz et al., 2012). For people who are prone to problem gambling, there is no scientific evidence to date to support a reduction in problematic behavior and depressive symptoms. Since depressive disorders often precede pathological gambling behavior (e. g. Blaszczynski & Nower, 2002; Lorains et al., 2011), the effectiveness of Deprexis in pathological gambling will be investigated in the present study.

(…)

4) Wait-list control group

The wait-list control group receives the training program at the end of the post assessment (i. e. after eight weeks).

(…)

**5. Information on the recording, processing, storage and deletion of data**

**5.1 Personal data**

All participants are advised that the data collected in this study will be processed and published anonymously. The anonymity of the participants is guaranteed, especially since the non-anonymous investigators can be contacted via email in case of questions (for information on anonymous email address, see below). Thereby it is possible to support the participants without their identification. The entries are automatically registered when online-questionnaires are completed and can be downloaded directly as a SPSS file. *IP addresses are not stored.* Names and address data (including state) are not requested. In order to compare the groups with regard to important socio-demographic data, the gender and age of the participants are surveyed. In order to be able to differentiate whether the effectiveness of one of the two programs is influenced by the medication of the participants, the current drug intake is also investigated. No further personal data is collected. At the end of the online survey, participants enter a code word so that the data collected in the post survey can be assigned to the pre-data. The code word is created according to the criteria you suggest. Participants receive instructions on how to create an anonymous e-mail address that does not reveal their identity (e. g. sunflower83@gmx.de instead of hans.meyer83@gmx.de).

**5.2 Data protection**

The evaluation and analysis of the data will be made in anonymous form by the study investigator and his colleagues. The data collected in the course of the study will only be passed on anonymously. The same applies to the publication of data. All collected data is stored on password-protected computers known only to the respective computer user. Personal data is stored on computers that do not have an Internet connection. CD-ROMs for hard disk storage are stored in a safe, the code of the safe is only known to the department's permanent staff. Our clinic is certified and the safety standards are regularly monitored by Germanischer Lloyd and interim internal audits. All project members are trained in this matter. If participants express their willingness to participate in further studies, we obtain a written declaration of consent that can be revoked at any time and we will only use this data internally. Data is not passed on to third parties.

(…)

**6.4 Inclusion and exclusion criteria**

**Inclusion criteria:**

- Age: 18 to 65 years old
- Consent to participate in study (informed consent)
- Internet access
- Sufficient command of the German language
- Willingness to participate in two anonymous online surveys, each of which lasts approx. 25-30 minutes
- Willingness to participate in an 8-week training program
- Willingness to use the program on one's own responsibility

- Willingness to provide an e-mail address
- Presence of at least moderately pronounced depressive symptoms

**Exclusion criteria:**

- Presence of life-time schizophrenia or bipolar disorder
- Presence of acute suicidal tendencies

(…)
